# Comprehensive analyses of immune activity in COVID-19-vaccinated idiopathic pulmonary fibrosis patients

**DOI:** 10.3389/fimmu.2024.1436491

**Published:** 2025-01-08

**Authors:** Agata Maciejewska, Piotr Czernia, Magdalena Piotrowska-Mieczkowska, Beata Wajda, Bartosz Słomiński, Jan Romantowski, Adam Sudoł, Małgorzata Dąbrowska, Lucyna Górska, Tomasz Smiatacz, Marek Niedoszytko, Ewa Jassem, Maria Skrzypkowska, Piotr Trzonkowski

**Affiliations:** ^1^ Department of Medical Immunology, Medical University of Gdansk, Gdansk, Poland; ^2^ Department of Pneumonology, Medical University of Gdansk, Gdansk, Poland; ^3^ Department of Allergology, Medical University of Gdansk, Gdansk, Poland; ^4^ Central Clinical Laboratory, University Clinical Centre, Gdansk, Poland; ^5^ Department of Infectious Diseases, Medical University of Gdansk, Gdansk, Poland

**Keywords:** IPF, idiopathic pulmonary fibrosis, phenotype, flow cytometry, antibodies, cytokines, COVID-19, vaccine

## Abstract

Idiopathic pulmonary fibrosis (IPF) is a progressive and fatal disease, characterized by impaired wound repair, tissue remodeling and fibrosis. Immune system may participate in the development and progression of the disease as indicated by altered activity in IPF sufferers. This study investigates the immune response to the BNT162b2 COVID-19 vaccine in patients with IPF compared to healthy controls, with a particular focus on evaluation of antibody responses, interferon-gamma release, cytokine profiling and a broad panel of immune cell subpopulations. IPF patients without prior exposure to SARS-CoV-2 had undetectable levels of anti-N IgG antibodies, highlighting their lack of previous infection. After vaccination, IPF patients showed a significant increase in anti-S1 IgG and IgA antibodies, though their levels were lower compared to healthy controls and convalescent IPF patients. Additionally, IPF patients exhibited altered proportions of regulatory T cells (Tregs) and effector T lymphocytes (Teffs) before and after vaccination. Specifically, IPF patients had higher percentages of Tregs with a Th2 phenotype and Th17 Tregs, along with reduced proportions of Th1/17 Tregs. Teffs in IPF patients showed a decrease in Th1-like and Th2-like populations after vaccination. Moreover, IPF patients demonstrated elevated populations of cytotoxic T lymphocytes (Tc) before vaccination and increased levels of γδ Tc cells throughout the study. Alterations in cytokine profiles were also observed, IPF patients showed higher levels of IL-6 and IL-22 compared to healthy controls. These findings suggest a distinct immune response in IPF patients to the COVID-19 vaccine, characterized by differences in antibody production, T cell differentiation and cytokine secretion compared to healthy individuals.

## 1 Introduction

Idiopathic pulmonary fibrosis (IPF) is a primary, progressive and fatal disease with an average life expectancy between 3 and 5 years after diagnosis (1). It is hypothesized to develop due to repetitive epithelial damage that leads to persistent inflammation, impaired wound repair and tissue remodeling, ultimately followed by fibrosis (2). Even though the impaired activation of alveolar epithelial cells (AECs) as well as accumulation of extracellular matrix-producing fibroblasts and myofibroblasts are considered the most characteristic features of IPF pathogenesis, the exact etiology of this disease has not been determined (2, 3). Currently, the only available pharmacological treatment of IPF consists of two antifibrotics, pirfenidone and nintedanib, which reduce the progression rate (4).

Disappointing results from clinical trials (5) have led to redirection from the inflammation basis of the IPF hypothesis. However, the immune system could still be considered involved in the development and/or progression of the disease, as the discouraging effects of immunotherapies may simply result from incorrect selection of immunomodulators (6). The immune system exhibits altered activity in IPF sufferers, with both innate and adaptive responses being engaged in fibrosis (2). Fibrosis development as well as its severity have been linked to immune-relevant genes (7, 8).

Refined knowledge of the immune system’s participation in IPF could facilitate the development of therapies to improve current treatment. Blood sample-based analyses are being designed to create easily accessible biomarkers of disease progression (6). Recent studies using single-cell RNA sequencing (scRNA-seq) have shown that changes in immune cell populations and signaling pathways can drive the progression of IPF. Understanding these alterations is crucial for identifying potential therapeutic targets to manage or slow down the disease (9, 10).

Severe acute respiratory syndrome coronavirus 2 (SARS-CoV-2) is an enveloped +ssRNA virus that belongs to the Coronaviridae family and is responsible for Coronavirus disease 2019 (COVID-19) (11). Due to easy transmission approximately 774,291,287 cases of SARS-CoV-2 infection worldwide and 7,019,704 deaths have been confirmed by the World Health Organization (WHO) by January 2024 (12). As a result of therapy and vaccine development, SARS-CoV-2 infections have become recognized as common and controllable disease. However, given its impact on respiratory status in patients with chronic lung diseases, it remains a concern for pulmonologists (13).

BNT162b2 is a lipid nanoparticle–formulated, nucleoside-modified RNA vaccine that encodes a perfusion-stabilized, membrane-anchored SARS-CoV-2 full-length spike (S) protein (14). The efficacy and safety of the vaccine against laboratory-confirmed COVID-19 have been proven (15, 16). High protection against symptomatic COVID-19 as well as severe form of the disease, need for hospitalization and death have also been confirmed (17, 18).

Patients with IPF are much more likely to experience disease exacerbation if suffering from pulmonary comorbidities or simple viral infection. Analyses of data concerning patients with interstitial lung diseases (ILD) suggest that IPF is associated with poor clinical outcomes of COVID-19 (19). Therefore, one may expect that vaccination against SARS-CoV-2 in this population could protect against disease deterioration and complication development (20). Previously, initial immunization of IPF patients against SARS-CoV-2 has raised some concerns (21, 22). According to our knowledge, this is the first publication describing the effect of the COVID-19 vaccine in this group to such extend.

The aim of our study was to explore alterations in the response to the BNT162b2 mRNA COVID-19 vaccine in IPF patients compared to healthy volunteers. Therefore, firstly, we have analyzed the production of SARS-CoV-2 anti-S antibodies of the IgG and IgA classes after vaccination. We have also evaluated interferon (IFN) γ release upon SARS-Cov-2 spike protein stimulation in cells isolated from subjects before and after vaccination. We have also determined the levels of selected cytokines, namely: interleukin (IL)-6, IL-17, IL-22, tumor necrosis factor alpha (TNF- α) and transforming growth factor beta-1 (TGF-β1) in blood serum samples. Finally, we have performed extensive analyses of the phenotypic profiles of B, T and NK lymphocytes.

## 2 Materials and methods

### 2.1 Study design

24 Polish individuals (aged 52-76), diagnosed with IPF according to the 2011 international diagnostic guidelines (23) have been enrolled in our study. All patients remained under the care of the Clinic of Pneumonology at the University Clinical Centre in Gdansk, who were qualified for the drug program (pirfenidone/nintedanib therapy). IPF patients were considered a high-risk group and therefore were referred for vaccination against SARS-CoV-2. The control group included 29 healthy volunteers (aged 40-71), undergoing SARS-CoV-2 vaccination.

Patients with prior exposure to the SARS-CoV-2 virus have been identified by medical history, interview and the level of anti-N protein IgG antibodies before vaccination. Individuals who underwent infection were considered convalescents and were analyzed separately. Ultimately, 7 participants in the IPF group and 8 volunteers in the control group were recognized as previously infected.

Written informed consents were obtained from all participants. The study was approved by the Ethics Committee of the Medical University of Gdańsk (NKBBN/243/2021), written informed consent was obtained from all participants and our investigation was carried out in accordance with the Code of 8 Ethics of the World Medical Association (Declaration of Helsinki) for experiments on human subjects.

### 2.2 Sample collection and preparation

First, venous blood samples were collected in EDTA-containing tubes at three time points: before vaccination, 6 weeks after the 1^st^ dose (immediately before the second dose), and 14 days after the 2^nd^ dose. Peripheral blood mononuclear cells (PBMCs) were isolated by Ficoll-Paque™Plus (Cytiva, MA, USA) gradient centrifugation. Cell number and viability were assessed using a Bio-Rad TC20™ cell counter. Isolated PBMCs were cryopreserved in liquid nitrogen for further analyses. To isolate the serum, venous blood was also collected into clot activator-containing tubes. After 30 minutes of incubation, the tubes were centrifuged for 15 minutes at 1000 × g. Serum was collected, aliquoted and stored at -80°C until further analyses.

### 2.3 Anti-SARS-CoV-2 IgG antibody testing

#### 2.3.1 Anti-N

To determine the occurrence of previous COVID-19 infection the levels of IgG antibodies against the SARS-CoV-2 nucleocapsid antigen were detected, using the Abbot Architect™ SARS-CoV-2 IgG assay according to the manufacturer’s protocol. To enable comparisons of results between different laboratories using binding affinity units/milliliter (BAU/ml), a conversion factor of 0.142 was applied.

#### 2.3.2 Anti-S

The DiaSorin LIAISON^®^SARS-CoV-2 S1/S2 IgG serological test was used to detect the concentration of neutralizing SARS-CoV-2 anti-S (S1 and S2 subunits) IgG antibodies. Seroconversion after vaccination was set at 39 BAU/ml of anti-S1 IgG antibodies. To enable comparisons of results between different laboratories using BAU/ml, a conversion factor of 2.6 was applied.

### 2.4 Anti-SARS-CoV-2 IgA antibody testing

#### 2.4.1 Anti-S

The quantitative measurements of SARS-CoV-2 anti-S1 receptor binding domain (RDB) IgA antibodies were performed using a COVID-19 S-Protein (S1RBD) Human IgA ELISA Kit (Abcam, UK) according to the manufacturer’s protocol.

### 2.5 IFNγ release assay (IGRA test)

PBMCs were used to analyze IFNγ release upon S1 SARS-CoV-2 spike protein stimulation. Thawed cells were cultured for 24 h before the test. Cell viability was evaluated and samples with values below 70% were excluded. PBMCs were stimulated with a SARS-CoV-2 IGRA stimulation tube set (Euroimmun Medizinische Labordiagnostika AG, Germany) according to the manufacturer’s instructions. Cytokine release was evaluated using the interferon-gamma ELISA set (Euroimmun Medizinische Labordiagnostika AG, Germany). The threshold of IFNγ release was set based on cytokine secretion characteristics for unvaccinated individuals.

### 2.6 Flow cytometry analysis

PBMCs were labeled with a specific panel of anti-human monoclonal antibodies targeting surface markers (**Supplementary Table 1; Supplementary Data**). The staining procedures were performed in accordance with the manufacturer’s recommendations. Flow cytometric analyses were conducted using the LSE Fortessa™ Cytometer (BD Bioscience, CA, USA). The flow cytometry gating strategy is illustrated in **Supplementary Figure 1** within the **Supplementary Data File**. The analyzed populations are listed in **Supplementary Table 2; Supplementary Data**.

### 2.7 Cytokines

The concentrations of interleukin 6 (IL-6), interleukin 17 (IL-17), interleukin 22 (IL-22), tumor necrosis factor alpha (TNF-α) and transforming growth factor beta-1 (TGF-β1) were evaluated in serum samples using a Human IL-6 Quantikine ELISA kit, cat. no D6050; a Human IL-17 Quantikine ELISA kit, cat. no D1700; a Human IL-22 Quantikine ELISA kit, cat. no D2200; a Human TNF-α Quantikine ELISA kit, cat. no DTA00D and a Human/Mouse/Rat/Porcine/Canine TGF-β1 Quantikine ELISA kit, cat. no DB100C (R&D Systems Inc., MN, USA) according to the manufacturer’s protocol.

### 2.8 Statistical analysis

The results were analyzed using Statistica, version 13.3 (StatSoft Inc, OK, USA). The distribution of parameters was tested using the Shapiro-Wilk test. Most of the parameters were characterized by a nonnormal distribution; therefore, the Mann-Whitney U test was used to assess independent continuous data. For parameters with a normal distribution, Levene’s test of homogeneity of variance was performed. To analyze the increase in antibodies in response to vaccination, the Wilcoxon signed-rank test for paired observations was applied. The data are presented as medians with interquartile ranges. Significant results are marked with * (p<0.05), ** (p<0.01) or *** (p<0.001). To verify differences in IPF and control individuals, Principal Component Analysis (PCA) was performed for all analyzed phenotypes at three time points (**Supplementary Data File**).

## 3 Results

### 3.1 Clinical characteristics of participants

The brief characteristic of distinguished groups is shown in **Table 1**.

**Table 1:** The characteristic of patients included into the study. The data are presented as medians (IQRs).

	IPF	IPF conv.^#^	Control	Control conv.^#^
N	17	7	21	8
Female: Male	04:13	03:04	07:14	00:08
Age	68 (63-71)*	66 (62-68)	55 (49-70)*	51 (49-62)
Antifibrotic			N/A	N/A
Pirfenidone	7	2		
Nintedanib	9	5		
None	1	–		
BMI	29 (27-35)	26 (24-27)	N/A	N/A
*Z-score^1^*	-4.18 (-4.34 – -3.71)	-4.83 (-5.37 – -3.50)	N/A	N/A

*The age difference between IPF patients and their healthy counterparts was significant with p<0.05.

^#^conv. – convalescent.

^1^The *z-score* is a parameter that indicates the efficiency of pulmonary gas exchange in the DLCO test. This test is performed during antifibrotic therapy as part of the national treatment program. Scores ≤ -4 indicate severe pulmonary dysfunction according to ATS/ERS 2021.

N/A, not available/not applicable.

A statistically significant age difference was observed between the IPF and the control patients (p=0.04). Sex distribution did not differ significantly between these groups (p=0.76).

The **Supplementary Material** includes a table with clinical data of the patients (**Supplementary Table 3; Supplementary Data**).

At the beginning of the study, all recruited subjects have been screened for the previous infection with SARS-CoV-2. As expected, IPF patients without previous contact with the virus had undetectable levels of anti-N IgG antibodies [0.03 BAU/ml (0.02-0.04)] compared to convalescent IPF [2.69 BAU/ml (1.09-5.86)] (p<0.001)].

### 3.2 Concentrations of anti-S1 IgG antibodies

Before vaccination, patients in IPF group and the control group had anti-S1 IgG antibody levels below the detection limit, which was 4.81 BAU/ml.

**Figure 1:**
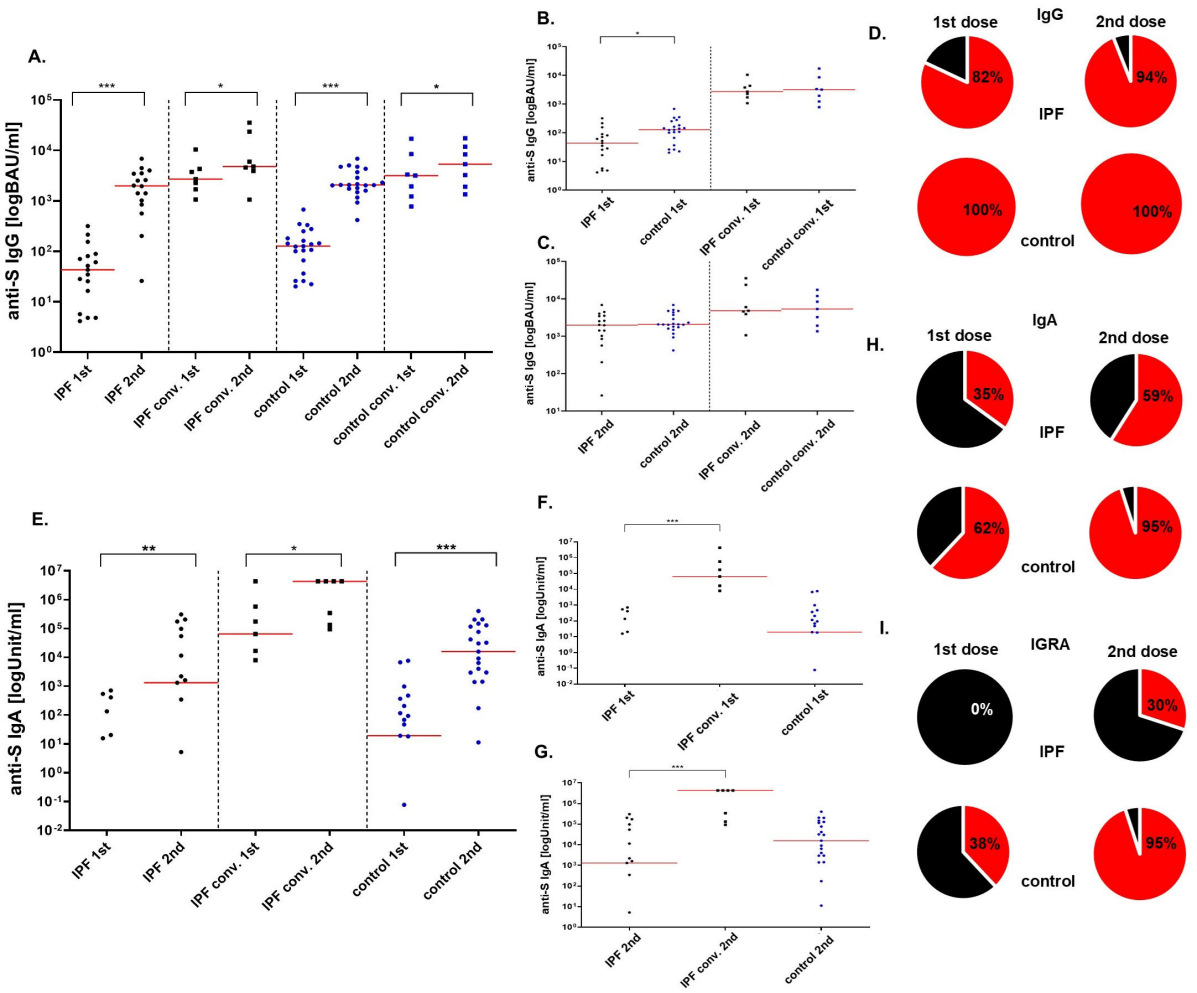
The levels of anti-S IgG (BAU/ml), anti-S IgA (Unit/ml) and the percentage of seroconversion rate after the BNT162b vaccine. Four sections based on the type of the group are shown: IPF, IPF convalescent, control, and control convalescent **(A)** Increase in anti-S IgG antibodies after the second dose of the vaccine in all analyzed groups. **(B)** Antibody levels after the first dose of vaccine in all groups after the first and the second **(C)** dose of vaccine. **(D)** On the left: seroconversion rate after the first dose, on the right: seroconversion rate after the second dose. The cut-off for the positive seroconversion rate for anti-S IgG was ≥39 BAU/ml. The circle divides patients into: responders (red—positive anti-S IgG titer after vaccine) and non-responders (black—patients without anti-S IgG). **(E)** Increase in anti-S IgA antibodies after the second dose of the vaccine in all analyzed groups. **(F)** Antibody levels after the first dose of vaccine in all groups after the first and the second **(G)** dose of vaccine. **(H)** On the left: seroconversion rate after the first dose, on the right: seroconversion rate after the second dose. The circle divides patients into: responders (red—positive anti-S IgA titer after vaccine) and non-responders (black—patients without anti-S IgA). INFγ secretion response **(I)** On the left: responders rate after the first dose, on the right: responders rate after the second dose. The circle divides patients into: responders (red— positive IFNγ secretion response after vaccine) and non-responders (black—patients without IFNγ secretion response). The red line indicates the median. Significant results are marked with * (p<0.05), ** (p<0.01), or *** (p<0.001); conv. – convalescent.

The seroconversion rate in IPF patients and healthy controls without prior exposure to SARS-CoV-2 was investigated. The rates of seroconversion achieved by the IPF group and healthy volunteers after the 1^st^ (82% vs 100%; p>0.05) and 2^nd^ (94% vs 100%, p>0.05) BNT162b2 vaccine doses were similar (**Figures 1A-D**).

In all groups, a significant increase in the concentration of anti-S1 IgG antibodies was observed between the 1^st^ and 2^nd^ courses of vaccination as anticipated (p<0.000 for IPF patients without previous exposure to SARS-CoV-2; p=0.03 for convalescent IPF sufferers; p<0.000 for the control group and p=0.02 for convalescent controls).

Furthermore, IPF patients without prior exposure to SARS-CoV-2 had lower concentrations of anti-S1 IgG antibodies after the 1^st^ dose of the BNT162b2 vaccine than did healthy individuals [43 BAU/ml (17-81) vs. 127 BAU/ml (66-182); p=0.011]. The levels of antibodies after the 2^nd^ dose of vaccine did not differ between the groups [1963 BAU/ml (848-3458) vs 2080 BAU/ml (1747-3718); p=0.212].

In contrast to control convalescents, convalescent IPF patients had similar concentrations of anti-S1 IgG antibodies after 1^st^ [2704 BAU/ml (1698-4316) vs. 3250 BAU/ml (1583-6240); p=0.95] and 2^nd^ [4810 BAU/ml (3926-23686) vs 6799 BAU/ml (2583-10140); p=0.95] vaccination (**Figures 1A-D**).

### 3.3 Concentrations of anti-S IgA antibodies

The generation of anti-S1 IgA antibodies after the immunization cycle in IPF patients and healthy controls was also investigated. In all analyzed groups, a significant increase in the concentration of anti-S1 IgA antibodies was observed between the 1^st^ and 2^nd^ course of vaccination (p=0.003 for IPF patients without previous exposure to SARS-CoV-2; p=0.0001 for the control group and p=0.03 for convalescent IPF sufferers), as expected. Due to the shortage of serum samples, IgA levels were not assessed in convalescent controls.

The IPF group produced antibodies less frequently after the 1^st^ (35%) and the 2^nd^ (59%) doses of the BNT162b2 vaccine when compared to healthy volunteers (62% and 95%, respectively). However, these differences did not reach statistical significance (p>0.05) (**Figures 1E-H**).

Differences in the concentrations of anti-S1 IgA antibodies after vaccination in IPF sufferers without prior exposure to SARS-CoV-2 and control group were not statistically significant [0 Units/milliliter (U/ml) (0-20) vs. 19 U/ml (0-206); p>0.05 after the 1^st^ dose; 1313 U/ml (0-54416) vs. 15829 U/ml (2970-116416); p>0.05 after the 2^nd^ dose]. However, antibodies concentrations in IPF patients without prior pathogen exposure were decreased when compared with convalescent IPF group [0 U/ml (0-20) vs. 64124 U/ml (1698-4316); p=0.001 after the 1^st^ dose; 1313 U/ml (0-54416) vs. 4328321U/ml (133809-4328321); p=0.001 after the 2^nd^ dose] (**Figures 1E-H**).

### 3.4 IFNγ release assay

To determine the cellular responses of patients after vaccination, IFNγ release by S1 protein-stimulated PBMCs has been investigated.

Cells isolated after the 1^st^ vaccination of IPF individuals without previous exposure to SARS-CoV-2 did not respond to stimulation (0%). The rate of response in healthy volunteers was significantly higher (p=0.003) and occurred in 38% of the subjects. The IFNγ secretion response improved after the 2^nd^ vaccine dose but remained lower in the IPF group when compared to controls (30% vs 95%; p=0.0001) (**Figure 1I**).

The concentrations of released cytokine were comparable between the groups (data not shown).

### 3.5 Flow cytometry analysis

#### 3.5.1 Altered differentiation of regulatory T lymphocytes in IPF

**Figure 2:**
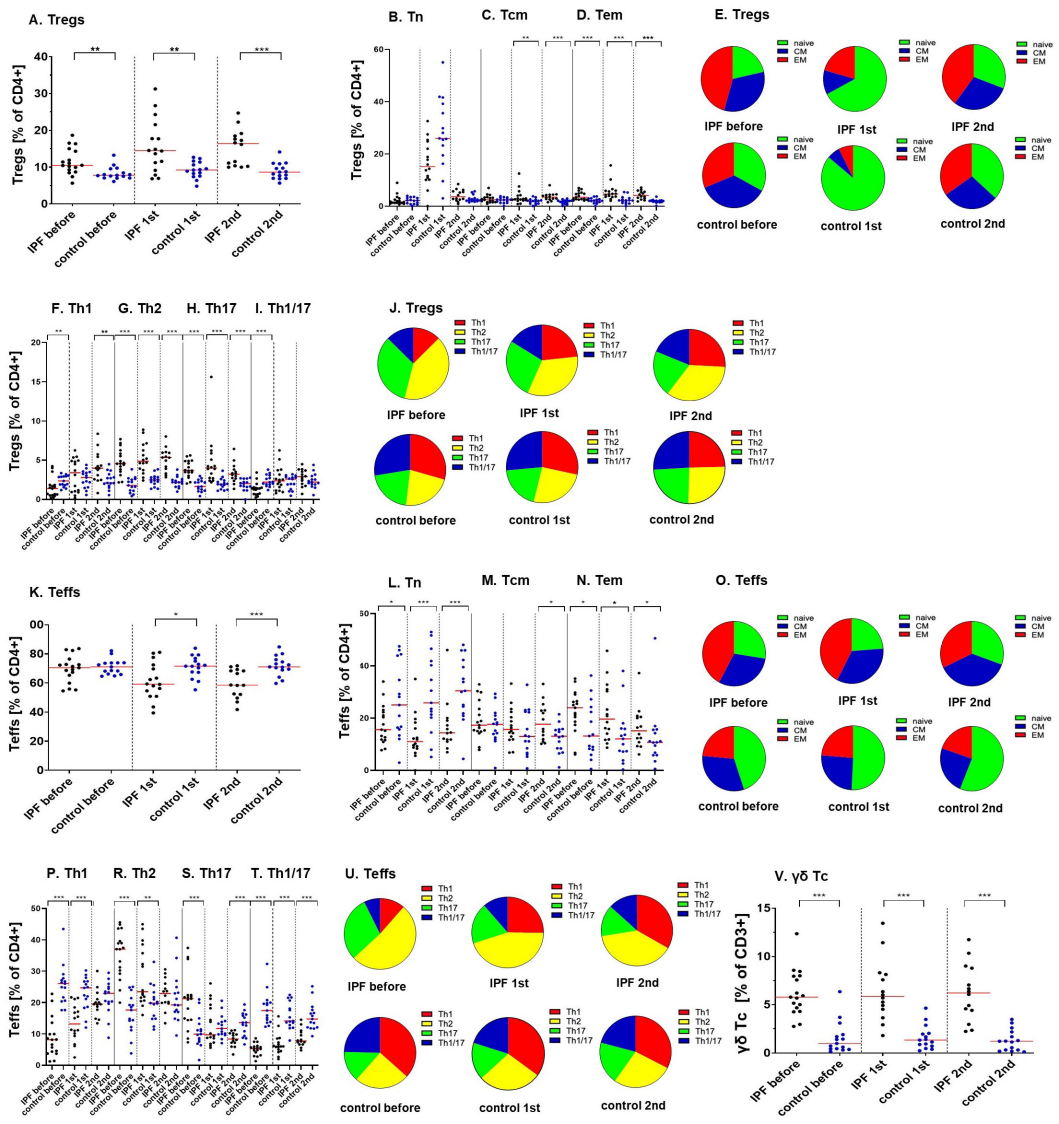
Ratios of various regulatory, effector and cytotoxic T lymphocytes populations during the vaccination course (before, after the 1^st^ and the 2^nd^ vaccine dose) between analyzed groups (IPF vs. healthy control). **(A)** Tregs; **(B)** Tn, **(C)** Tcm, **(D)** Tem Tregs; **(E)** distribution of Tn, Tcm, Tem Tregs; **(F)** Th1, **(G)** Th2, **(H)** Th17, **(I)** Th1/17 Tregs; **(J)** distribution of Th1, Th2, Th17, Th1/17 Tregs; **(K)** Teffs; **(L)** Tn, **(M)** Tcm, **(N)** Tem Teffs; **(O)** distribution of Tn, Tcm, Tem Teffs; **(P)** Th1, **(R)** Th2, **(S)** Th17, **(T)** Th1/17 Teffs; **(U)** distribution of Th1, Th2, Th17, Th1/17 Teffs; **(V)** γδ cytotoxic T cells. The red line indicates the median. Significant results are marked with *(p<0.05), **(p<0.01), or ***(p<0.001).

We have observed increased percentages of regulatory T cells (Tregs) within the CD4+ population in IPF patients when compared to healthy controls (10.51% vs 7.78%; p=0.002 before vaccination; 14.46% vs 9.23%; p=0.006 after the 1^st^ dose; 16.40% vs 8.65%; p<0.000 after the 2^nd^ dose of vaccine) (**Figure 2A**) (PCA 1-3; **Supplementary Table 4; Supplementary Data**).

We have determined the expression of classical differentiation markers on Tregs. The percentages of central memory (CM) Tregs did not differ between groups before vaccination (2.47% vs 2.40%; p=0.48) but increased in IPF sufferers after vaccination (2.72% vs 2.01%; p=0.01 after the 1^st^ dose; 3.48% vs 1.84%; p<0.000 after the second dose). The proportions of effector memory (EM) Tregs were heightened in patients at all established time points (3.43% vs 2.08%; p=0.001 before vaccination; 4.66% vs 2.21%; p<0.000 after the 1^st^ dose; 4.09% vs 2.02%; p<0.000 after the 2^nd^ dose) (**Figures 2B-E**)(**Supplementary Table 4**, PCA 1-3; **Supplementary Data**).

#### 3.5.2 Effect of IPF on Tregs phenotype during differentiation

We have observed a significant impact of IPF on the differentiation of Tregs with established phenotypes.

In comparison to control subjects, the proportions of Tregs that displayed a Th1 phenotype were decreased in IPF patients before vaccination procedure (1.39% vs 2.35%; p=0.01) but increased in this group 2 weeks after the 2^nd^ vaccine dose was administrated (3.99% vs 2.10%; p=0.008). Vaccination was associated with increased ratios of Th1 Tregs (p=0.001) in the IPF group. The prevalence of CM Th1 Tregs was lowered in IPF patients (0.21% vs 0.68%; p=0.001 before vaccination; 0.99% vs 0.49%; p= 0.004 after the 2^nd^ vaccine dose). The proportions of cells also increased in IPF after vaccination (p=0.001). Similarly, compared with healthy controls, the percentages of EM Th1-like Tregs in IPF patients were decreased before (0.34% vs 0.55%; p=0.03) but increased after vaccination (0.91% vs 0.46%; p=0.001). Analysis of variance suggested an increase in the number of cells in IPF patients after vaccination (p<0.000).

Our results demonstrated that IPF patients are characterized by significantly higher proportions of Tregs with the Th2 phenotype (4.56% vs 1.79%; p<0.000 before vaccination; 4.87% vs 2.49%; p<0.000 after the 1^st^ dose; 5.34% vs 2.20%; p<0.000 after the 2^nd^ dose) when compared to healthy individuals. This group also had increased proportions of CM Th2 cells (1.36% vs 0.75%; p=0.004 before vaccination; 1.13% vs 0.47%; p=0.004 after the 1^st^ dose; 1.39% vs 0.56%; p<0.000 after the 2^nd^ dose) as well as EM Th2 cells (1.70% vs 0.54%; p<0.000 before vaccination; 1.99% vs 0.65%; p<0.000 after the 1^st^ dose; 1.40% vs 0.53%; p<0.000 after the 2^nd^ dose).

Similarly, the ratios of Tregs that display features of Th17 population were increased in IPF patients (3.68% vs 1.64%; p<0.000 before vaccination; 3.99% vs 1.93%; p<0.000 after the 1^st^ dose; 3.22% vs 2.02%; p<0.000 after the 2^nd^ dose) when contrasted with control subjects. IPF sufferers displayed significant increases in: CM Th17 cells (0.80% vs 0.42%; p=0.04 before vaccination; 0.67% vs 0.30%; p=0.07 after the 1^st^ dose; 0.55% vs 0.28%; p=0.003 after the 2^nd^ dose) and EM Th17 Tregs (1.44% vs 0.43%; p<0.000 before vaccination; 1.29% vs 0.48%; p<0.000 after the 1^st^ dose; 0.88% vs 0.42%; p<0.000 after the 2^nd^ dose).

The percentages of Th1/17 Tregs subpopulation within CD4+ cells were significantly lower in IPF patients, than in healthy volunteers before vaccination (1.39% vs 2.20%; p<0.000), but not after the vaccine administration (2.36% vs 2.59%; p=0.83; and 2.90% vs 2.21%; p=0.20). Simultaneously, in IPF the percentages of Th1/17 Tregs increased after vaccination (p<0.000). The results for central and effector memory Th1/17 Tregs suggest decreased lymphocyte proportions in IPF patients before vaccination (0.13% vs 0.37%; p=0.001 for EM cells; 0.30% vs 0.49%; p=0.01 for CM cells) and heightened ratios after vaccine administration (0.44% vs 0.31%; p=0.05 for EM cells; 0.77% vs 0.43%; p=0.03 for CM cells) when compared to control group (**Figures 2F-J**) (**Supplementary Table 5**; PCA 1-3; **Supplementary Data**).

Additionally, we have analyzed cells that co-express inducible T cell co-stimulator (ICOS) and programmed cell death protein 1 (PD-1) receptors. Before the vaccine administration, the percentages of ICOS+PD-1+ Tregs within the CD4+ population were significantly decreased in IPF patients (0.33% vs 0.64%; p=0.02) when compared to control.

Further analyses of ICOS+PD-1+ and follicular Tregs are presented in the **Supplementary Materials** (Supplementary results; Heat map 1., **Supplementary Data File**).

#### 3.5.3 Impact of IPF on effector T lymphocytes maturation

The ratios of effector T lymphocytes (Teffs) within the T helper population were decreased in IPF after vaccination (59.19% vs 71.58% after the 1^st^ dose; p=0.02; 58.51% vs 71.06%; p=0.001 after the 2^nd^ dose). Analysis of variance revealed a significant reduction in the Teffs/CD4+ ratios in patients after vaccination (p=0.001). We have also observed heightened Tregs/Teffs ratios in IPF patients when compared to control group at all three time points (0.16% vs 0.11%; p=0.007 before vaccination; 0.24% vs 0.13%; p=0.007 after the 1st dose; 0.28% vs 0.12%; p<0.000 after the 2nd dose) (**Figure 2K**). Moreover, the Tregs/Teffs proportions were increased in IPF after vaccination (p=0.02) (**Supplementary Table 6**; PCA 4-6; **Supplementary Data**).

We have also analyzed the impact of IPF on the expression of maturation-related markers of effector T lymphocytes. The percentages of naïve Teffs within the CD4+ cell population were decreased in IPF (15.59% vs 25.04%; p= 0.04 before vaccination; 11.03% vs 25.85%; p=0.001 after the 1^st^ dose; 14.36% vs 30.45%; p=0.001 after the 2^nd^ dose). There was a significant increase in CM effector T lymphocytes population in IPF patients after the 2^nd^ vaccine dose was administered (17.68% vs 13.06%; p=0.03). The ratios of EM effector T cells have increased in IPF sufferers (23.87% vs 2.67%; p=0.04 before vaccination; 19.63% vs 12.07%; p=0.02 after the 1^st^ dose; 15.00% vs 10.69%; p=0.05 after the 2^nd^ dose) (**Figures 2L-O**) (**Supplementary Table 6**; PCA 4-6; **Supplementary Data**).

#### 3.5.4 Imbalance of Teffs subpopulations in IPF, different response to vaccination in IPF

The ratios of Th1 effector lymphocytes in IPF sufferers were lowered when compared to control group (8.15% vs 26.06%; p<0.000 before vaccination; 13.19% vs 24.70%; p<0.000 after the 1^st^ dose). The proportions of these cells decreased in healthy volunteers after vaccination (p=0.04). CM and EM Th1 Teffs were reduced in IPF before vaccination (1.67% vs 5.12%; p<0.000 for CM cells; 1.44% vs 4.43%; p=0.004 for EM cells) but increased after the procedure (6.16% vs 2.54%; p=0.001 for CM cells; 4.85% vs 3.00%; p=0.004 for EM cells). Ratios of these cells raised in IPF group (p=0.001 for CM; p<0.000 for EM) and declined healthy subjects (p=0.02 for CM; p=0.001 for EM) after vaccination.

Our results demonstrate that IPF patients are characterized by significantly higher proportions of Th2-like effector T lymphocytes during initial time points (37.05% vs 17.62%; p<0.000 before vaccination; 23.47% vs 19.83%; p=0.008 after the 1^st^ dose), but not after vaccination procedure completion (22.89% vs 19.26%; p=0.31). Vaccination was connected with significant decrease of Th2 effector T cells in IPF (p=0.008). Proportions of central and effector memory Th2 lymphocytes were heightened in IPF patients at all time points (for CM cells: 11.45% vs 5.06%; p<0.000 before vaccination; 7.84% vs 5.17%; p=0.01 after the 1^st^ dose; 7.90% vs 4.98%; p=0.02 after the 2^nd^ dose; for EM cells: 11.94% vs 2.50%; p<0.000 before vaccination; 8.08% vs 3.41%; p=0.001 after the 1^st^ dose; 4.53% vs 3.07%; p=0.06 after the 2^nd^ dose).

Results observed for Th17 Teffs were much more ambiguous - cells were increased in patients before vaccination (21.24 vs 9.94%; p=0.0007) but decreased when analyzing groups after procedure completion (8.36% vs 13.60%; p=0.001). Analysis of variance confirmed that the Th17 Teffs/CD4+ cell ratio was reduced in the IPF group after vaccination (p=0.001). Before the vaccine administration, the IPF group displayed significant increase of CM and EM Th17 cells, when compared to controls (4.36% vs 2.54%; p=0.02 for CM cells; 8.36% vs 1.72%; p<0.000 for EM cells, respectively). The proportions of effector memory Th17 cells were reduced in IPF sufferers after vaccination (p=0.02).

We have observed decreased ratios of Th-1/17 effector T cells in IPF patients at all time points (5.22% vs 17.49%; p<0.000 before vaccination; 5.94% vs 14.16%; p<0.000 after the 1^st^ dose; 7.76% vs 14.76%; p<0.000 after the 2^nd^ dose), even though the proportions of cells increased in IPF after vaccination (p=0.02). We have observed lowered ratios of CM Th1/17 cells in IPF at the initial time points (0.71% vs 2.87%; p<0.000 before vaccination; 1.07% vs 1.60%; p=0.05 after the 1^st^ dose), but not after vaccination completion (1.35% vs 1.39%; p=0.43). The proportions of cells were increased in the IPF group (p=0.000) but decreased in the control group (p=0.02) after vaccination. The proportions of EM Th1/17 cells were significantly decreased in IPF patients before vaccination procedure (1.38% vs 3.05%; p=0.004). Cells were significantly increased in IPF sufferers (p=0.02) but decreased in healthy subjects (p=0.001) after vaccination (**Figures 2P-U**)(**Supplementary Table 7**; PCA 4-6; **Supplementary Data**).

Analyses of follicular Teffs are presented in the supplemental materials (Supplementary results; Heat map 2., **Supplementary Table 7**; PCA 4-6; **Supplementary Data File**).

The results of helper T cells analyses are presented in **Supplementary Materials** (Supplementary results; Heat map 3., **Supplementary Table 8**; PCA 7-9; **Supplementary Data File**).

#### 3.5.5 Increased populations of cytotoxic T lymphocytes in IPF

The proportions of cytotoxic T cells (Tc) were significantly higher when analyzing IPF patients and healthy volunteers (23.68% vs 12.51%; p=0.0001 before vaccination; 32.79% vs 12.58%; p=0.0002 after the 1st dose; 22.45% vs 13.24%; p=0.002 after the 2^nd^ dose). What is more, the ratios of Th/Tc were lowered in IPF subjects (2.18% vs 5.01%; p=0.009 before vaccination; 1.55% vs 4.90%; p=0.002 after the 1^st^ dose; 1.87% vs 4.29%; p=0.01 after the 2^nd^ dose). Further analyses suggested increased proportions of truly naïve Tc cells in IPF patients (1.23% vs 0.66%; p=0.02 before vaccination; 0.86% vs 0.23%; p=0.02 after the 2^nd^ dose). Simultaneously, ANOVA testing revealed a decrease of cells’ proportions in IPF after vaccination (p=0.02). On the other hand, the ratios of naïve Tc/CD3+ were similar between the groups (2.87% vs 2.73%; p=0.47 before vaccination; 4.19% vs 2.54%; p=0.19 after the 1^st^ dose; 3.63% vs 2.18%; p=0.20 after the 2^nd^ dose). The proportions of central memory (2.16% vs 0.49%; p<0.000 before vaccination; 2.47% vs 0.71%; p<0.000 after the 1^st^ dose; 2.80% vs 0.52%; p<0.000 after the 2^nd^ dose) as well as effector memory (6.53% vs 0.78%; p<0.000 before vaccination; 7.28% vs 1.07%; p<0.000 after the 1^st^ dose; 6.32% vs 1.05%; p<0.000 after the 2^nd^ dose) cytotoxic T lymphocytes were increased in IPF patients when compared to control group. Analysis of terminally-differentiated CD45 RA-positive effector memory Tc cells revealed lack of significant differences between the groups (11.65% vs 6.19%; p=0.05 before vaccination; 14.64% vs 6.80%; p=0.29 after the 2^nd^ dose) (Heat map 4., **Supplementary Table 9**; PCA 10-12; **Supplementary Data**).

#### 3.5.6 Differentiation/activation and senescence related markers on Tc cells in IPF

We have also analyzed several markers connected with T cells’ differentiation and senescence. The prevalence of CD27+ Tc cells was heightened in IPF when compared to healthy subjects only before vaccination (2.64% vs 0.61%; p=0.002 before vaccination; 0.31% vs 0.20%; p=0.43 after the 1^st^ dose; 0.26% vs 0.14%; p=0.13 after the 2^nd^ dose). The proportions of cells decreased in the IPF group after vaccination (p=0.001). Analogous results have been observed for CD27-positive Tc cells during all stages of differentiation – before vaccination, the ratios of: naïve (0.46% vs 0.16%; p=0.03), CM (0.29% vs 0.06%; p=0.009), EM (0.39% vs 0.07%; p=0.003), as well as TEMRA (0.67% vs 0.21%; p=0.01) cells were higher in IPF patients when compared to healthy counterparts. The proportions were similar after vaccine administration (0.02% vs 0.07%; p=0.35 after the 1^st^ dose and 0.05% vs 0.04%; p=0.91 after the 2^nd^ dose for naïve cells; 0.08% vs 0.04%; p=0.31 after the 1^st^ dose and 0.09% vs 0.02%; p=0.08 after the 2^nd^ dose for CM cells; 0.08% vs 0.06%; p=0.40 after the 1^st^ dose and 0.07% vs 0.00%; p=0.07 after the 2^nd^ dose for EM cells; 0.16% vs 0.07%; p=0.25 after the 1^st^ dose and 0.12% vs 0.05%; p=0.08 after the 2^nd^ dose for TEMRA cells). After vaccination, the prevalence of naïve (p<0.000), CM (p<0.000), EM (p<0.000) as well as TEMRA (p=0.005) CD27+ Tc cells decreased in IPF. The proportions of central memory cells were also significantly reduced in healthy subjects (p=0.04) (Heat map 4., **Supplementary Table 10**; PCA 10-12; **Supplementary Data**).

The results obtained for CD27-, CD28+ and CD57+ cytotoxic T cells are presented in the **Supplementary Materials** (Supplementary results; **Supplementary Data File**).

Subsequent investigation of the senescent CD28-CD57+ Tc population revealed an increased ratio in IPF sufferers (14.03% vs 3.76%; p=0.003 before vaccination; 16.11% vs 3.93%; p=0.001 after the 1^st^ dose; 14.36% vs 4.46%; p=0.005 after the 2^nd^ dose). Analogous outcomes have been observed for: central memory (0.66% vs 0.10%; p<0.000 before vaccination; 0.71% vs 0.11%; p<0.000 after the 1^st^ dose; 0.69% vs 0.07%; p<0.000 after the 2^nd^ dose) or effector memory (2.65% vs 0.19%; p<0.000 before vaccination; 2.36% vs 0.30; p<0.000 after the 1^st^ dose; 2.38% vs 0.26%; p=0.0001 after the 2^nd^ dose) CD28-CD57+ cytotoxic T cells.

After the 1^st^ and 2^nd^ vaccine doses, the proportion of FasL+ Tc lymphocytes was lowered in the IPF group when compared to control group (4.02% vs 7.99; p=0.006 after the 1^st^ dose; 4.21% vs 12.98%; p=0.02 after the 2^nd^ dose) (Heat map 4., **Supplementary Table 10**; PCA 10-12; **Supplementary Data**).

The results of the MAIT CD8+ cells analyses are presented in the **Supplementary Materials** (Supplementary results, **Supplementary Table 10**; PCA 10-12; **Supplementary Data File**).

#### 3.5.7 γδ cytotoxic T cells in IPF

Patients in study group were characterized by higher proportions of overall γδ Tc lymphocytes within the CD3+ population (5.78% vs 0.98%; p<0.000 before vaccination; 5.86% vs 1.33%; p<0.000 after the 1^st^ dose; 6.22% vs 1.22%; p<0.000 after the 2^nd^ dose) (**Figure 2V**)(**Supplementary Table 11**; PCA 10-12; **Supplementary Data**).

IPF patients also displayed increased proportions of: recent thymic emigrants (0.31% vs 0.03%; p<0.000 before vaccination; 0.13% vs 0.02%; p=0.01 after the 2^nd^ dose), naïve (0.63% vs 0.28%; p=0.02 before vaccination; 0.79% vs 0.37%; p=0.03 after the 2^nd^ dose), central memory (0.44% vs 0.03%; p<0.000 before vaccination; 0.36% vs 0.05%; p<0.000 after the 1^st^ dose; 0.40% vs 0.04%; p<0.000 after the 2^nd^ dose), effector memory (1.38% vs 0.08%; p<0.000 before vaccination; 1.70% vs 0.10%; p<0.000 after the 1^st^ dose; 1.47% vs 0.06%; p<0.000 after the 2^nd^ dose) as well as TEMRA (2.76% vs 0.48%; p<0.000 before vaccination; 3.17% vs. 0.92%; p<0.000 after the 1^st^ dose; 3.33% vs 0.60%; p<0.000 after the 2^nd^ dose) γδ cytotoxic T lymphocytes cells.

The ratios of senescent CD28-CD57+ central memory (0.12% vs 0.02%; p<0.000 before vaccination; 0.12% vs 0.01%; p<0.00 after the 1^st^ dose; 0.19% vs 0.00%; p<0.000 after the 2^nd^ dose), effector memory (0.86% vs 0.02%; p<0.000 before vaccination; 0.81% vs 0.05%; p<0.000 after the 1^st^ dose; 0.78% vs 0.02%; p<0.000 after the 2^nd^ dose), as well as TEMRA γδ Tc (2.07% vs 0.26%; p<0.000 before vaccination; 2.18% vs 0.57%; p<0.000 after the 1^st^ dose; 2.38% vs 0.34%; p<0.000 after the 2^nd^ dose) cells were elevated in the IPF group (Heat map 4., **Supplementary Table 11**; PCA 10-12; **Supplementary Data**).

The results of the B and NK lymphocytes analyses are presented in the **Supplementary Materials** (Supplementary results; Heat map 5-6 **Supplementary Tables 12**, **13**; PCA 13-18; **Supplementary Data File**).

### 3.6 Alterations of cytokine profile in IPF patients

We have not recognized differences in the concentrations of TNF- α and TGF-β1 in the serum levels between any of the analyzed groups (data not shown). IL-17 levels in the serum samples of the enrolled participants were below minimum detectable concentration [15 picograms/milliliter (pg/ml)] (data not shown).

#### 3.6.1 Interleukin-6

**Figure 3:**
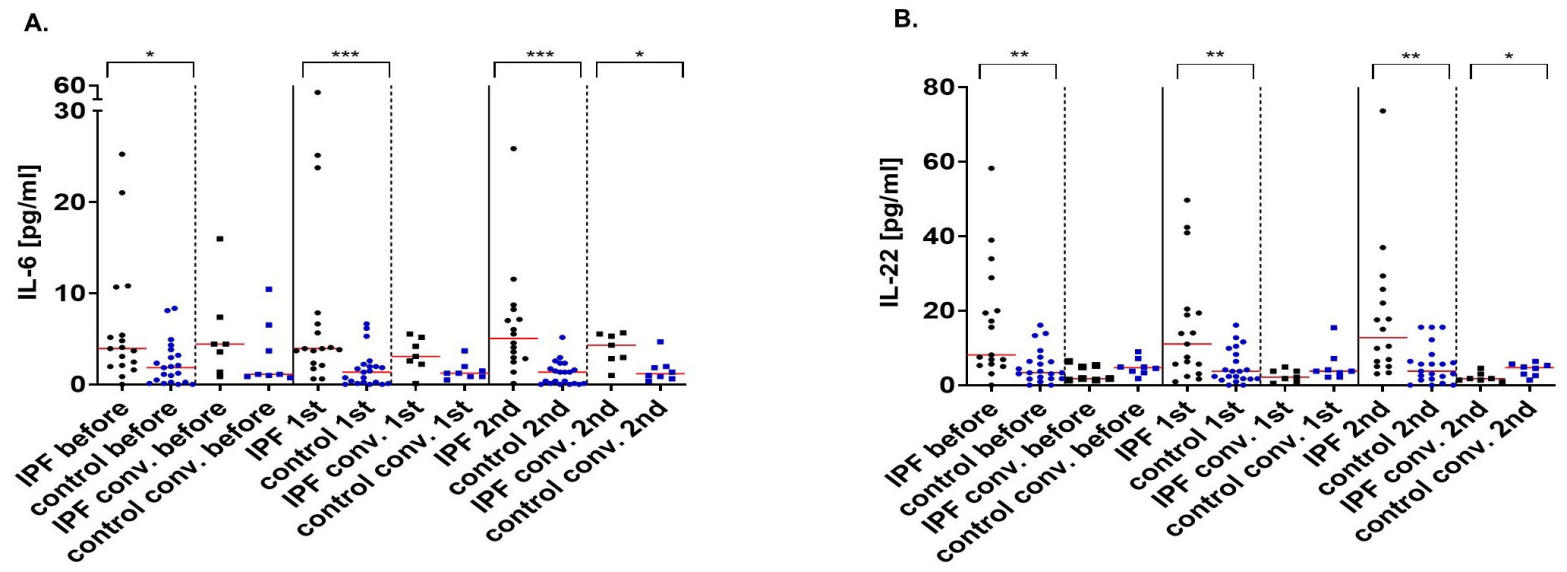
Interleukin-6 (pg/ml) **(A)** and interleukin-22 (pg/ml) **(B)** concentrations before, after the 1^st^ and 2^nd^ dose of the vaccine in IPF patients and healthy volunteers and their convalescent counterparts. The red line indicates the median. Significant results are marked with *(p<0.05), **(p<0.01), or ***(p<0.001). conv., convalescent.

IPF patients without prior exposure to SARS-CoV-2 exhibited higher concentrations of IL-6 when compared to healthy individuals before (3.93 pg/ml vs 1.84 pg/ml; p=0.016) and after vaccination (3.93 pg/ml vs 1.35 pg/ml; p<0.000 after the 1^st^ dose; 5.04 pg/ml vs 1.35 pg/ml; p<0.000 after the 2^nd^ dose]. Convalescent IPF patients and their control counterparts had similar concentrations of cytokine before (4 pg/ml vs 1.1 pg/ml; p=0.97) and after the first vaccine dose (3 pg/ml vs 1.23 pg/ml; p=0.056. However, the IPF group were characterized by heightened IL-6 levels after vaccination was complete (4 pg/ml vs 1.17 pg/ml; p=0.02) (**Figure 3A**).

#### 3.6.2 Interleukin-22

Compared with healthy subjects, IPF sufferers had higher concentrations of IL-22 when compared to healthy subjects (8.1 pg/ml vs 3.35 pg/ml; p=0.003 before vaccination; 11.04 pg/ml vs 3.72 pg/ml; p=0.01 after the 1^st^ dose; 12.73 pg/ml vs 3.74 pg/ml; p=0.0016 after the 2^nd^ dose). On the other hand, protein concentrations were comparable between the convalescent groups before (2 pg/ml vs 4.7 pg/ml; p=0.26) and after the first vaccination (2 pg/ml vs 3.74 pg/ml; p=0.14). After the 2^nd^ vaccine dose, IPF patients were characterized by lower IL-22 concentrations (2 pg/ml vs 4.7 pg/ml; p=0.03) (**Figure 3B**).

## 4 Discussion

The immune system plays an ambiguous role in idiopathic pulmonary fibrosis by simultaneously supporting protective and damaging processes (2). Altered immune responses in IPF sufferers should be expected during diseases or after vaccine administration. The response to vaccination may also be compromised due to therapy that patients with interstitial lung diseases (ILD) receive (24). The possibility of IPF exacerbation after vaccine administration should also be considered (22, 25). ILD have been indicated as a risk factor for adverse outcomes from SARS-CoV-2 infection - ILD patients display more than fourfold greater risk of death after COVID-19. This susceptible group is also in greater danger of more severe disease form, hospitalization and intensive care requirement (26, 27) with the highest risk of developing severe COVID-19 being recognized in IPF patients (27). This publication highlights the differences in numerous immune parameters between IPF patients and their healthy counterparts. Moreover, we investigated how distinguished groups had responded to BNT162b2 vaccine administration and what discrepancies could be identified.

IgG antibodies are significant for long-term immunity and provide systemic-wide defense (28). Our results suggest a delayed response to vaccination in IPF - despite observing similar anti-S1 IgG seroconversion between groups, IPF patients without previous SARS-CoV-2 exposure exhibited lower concentrations of antibodies after the 1^st^ vaccination. Pertzov et al. also recognized similar to healthy subjects IgG seroconversion rate and lower protein concentrations in IPF patients after two doses of the BNT162b2 vaccine. The authors concluded that this could result from ongoing disease as well as antifibrotic therapy (29). Significantly reduced IgG levels in BNT162b2-vaccinated IPF patients were also described by others (30). ILD has been indicated to be a risk factor for impaired IgG antibodies responses with defective interactions between leukocytes as responsible for poor vaccination outcomes (31).

Immunoglobulins A operate on mucosal surfaces that are the first entry point for the pathogens (32, 33). The participation of IgA alongside IgG and IgM antibodies in SARS-CoV-2 neutralization has been described (34) and the importance of secretory IgA induction in the prevention of COVID-19 development and spread has been postulated (35). Although, in our study, the differences in the seroconversion rate between patients and healthy subjects did not reach statistical significance, we hypothesize that analyses conducted on a larger population could reveal less frequent IgA-based responses in IPF. Similarly to general population (36), previous exposure to the SARS-CoV-2 virus increased antibodies secretion in patients.

Th-1 responses have been indicated to determine SARS-CoV-2 infection susceptibility (37) and protection after BNT162b2 vaccine administration (38). Low interferon γ serum levels were even distinguished as risk factor for COVID-19-caused lung fibrosis (39). Therefore, we have also determined the IFNγ secretion by S1 protein-stimulated PBMCs as a surrogate indicator of the cellular response. Cells isolated from IPF patients without prior SARS-CoV-2 infection did not respond after the 1^st^ dose of the BNT162b2 vaccine. Moreover, after the 2^nd^ vaccination, cytokine has been released by cells isolated from a significantly lower percentage of IPF patients when contrasted with healthy subjects. These results indicate compromised Th-1 responses in IPF patients after BNT162b2 vaccination and possibly impaired protection in this group as reduced IFNγ secretion has been connected with attenuated humoral responses to mRNA COVID-19 vaccines (40). The previously mentioned differences in the antibodies secretion in our IPF group could partially result from insufficient IFNγ production. To the best of our knowledge, this is the first study to evaluate IFNγ release by leukocytes of IPF patients after SARS-CoV-2 vaccine administration.

We have also analyzed the serum concentrations of several cytokines with IL-6 and IL-22 being elevated in IPF patients.

We have not observed any differences in TNF-α and TGF-β1 levels between the established groups. We were also unable to detect IL-17 in blood sera. Simultaneously, the concentrations of IL-6 and IL-22 were elevated in IPF patients without previous SARS-CoV-2 infection when compared to healthy volunteers – results indicate differences before as well as after vaccination. Moreover, the IL-22 concentration increased after each vaccination.

IL-6 has with fibrosis through, inter alia: promotion of fibroblast-to-myofibroblast transition (FMT) (41), blockage of fibroblasts apoptosis (42), collagen synthesis promotion or hyper-profibrotic macrophages development (43). Cytokine upregulation has been described in various fibrotic disorders including IPF (41). Increased levels of IL-6 transcripts in IPF-isolated lung tissue, heightened cytokine production by patients-isolated alveolar macrophages (44) or higher blood serum levels in patients with acute exacerbation IPF (45) have been reported. Serum protein concentrations have been distinguished as an indicator of exacerbation and poor prognosis in ILD (46) and IPF specifically (47). Our results indicate that a significant increase in the systemic IL-6 concentration could be detected even in stable, IPF patients receiving therapy.

IL-22 participates in connecting the pulmonary epithelium and immune system due to its role in pathogens clearance and epithelial regeneration (48). Cytokine protects epithelial cells from developing mesenchymal features and improves their viability after bleomycin administration (49). As expected, IL-22 deprivation worsens lung fibrosis (49). Whittington et al. described similar IL-22 concentrations in bronchoalveolar lavage fluid (BALF) samples from IPF and healthy subjects (50). It has been previously described as elevated in serum samples of lung cancer-associated IPF (51), asthma or pneumonia (52). Patients with uncomplicated SARS-CoV-2 infections are characterized by higher proportions of cytokine-producing lymphocytes (53). IL-22 also inhibits the expression of angiotensin-converting enzyme 2 (ACE2) as SARS-CoV-2 entry receptor (54). Taking into considerations our results, we believe that the protective effect of BNT162b2 mRNA COVID-19 vaccine via IL-22 induction is worth further investigation.

We have also focused on T cells with a regulatory phenotype. Despite the role of Tregs in IPF development has remined elusive, the hypothesis proposed by Boveda-Ruiz et al. states destructive activity of these cells during the early stages of the disease followed by protection as fibrosis progresses (55). Others have suggested that Tregs suppress initial inflammation in a CTLA-4-dependent manner but promote subsequent fibrosis by increasing IL-10 or TGF-β production (56). In studies using/utilizing/adopting single-cell RNA sequencing (scRNA-seq) (approach) for IPF significant changes in lymphocyte populations have been also identified. Findings include increased numbers of exhausted T cells with reduced functions, as well as defective regulatory T cells, which might contribute to the impairment of immune response in the fibrotic lung environment. These alterations have been associated with chronic inflammation and tissue remodeling in IPF, providing insights into potential therapeutic targets that aim to restore immune balance and reduce fibrosis progression (9). Some studies also noted dysregulation in B cell populations, potentially affecting antibody production and immune regulation (57).

Our IPF patients were characterized by increased proportions of Tregs, with effector memory cells being primarily responsible for these differences. Galati et al. also described heightened ratios of these cells but did not observe significant alterations in the proportions of naïve or memory Tregs between IPF and control subjects (58). Increased proportions of circulating Tregs have also been detected in newly recognized untreated IPF patients (59, 60). Others have reported a decreasing population of Tregs and their functional impairment in IPF (61). Tregs are known to undergo “specialization” in response to a specific environment - they differentiate alongside their respective effector counterparts as indicated by the expression of effector-specific chemokine receptors and transcription factors (62). Notably, Th-like Tregs are unlikely to exhibit a preference towards their respective effector (63). Tregs with a Th-like phenotype have the capacity to produce effector-specific cytokines such as: IFNγ (as Th1-like Tregs), IL-17 (as Th17-like Tregs), IL-4, IL-5 or IL-13 (as Th2-like Tregs) or IFNγ and IL-17 (as Th1/17-like Tregs) (63, 64).

Th2-like Tregs has been described as possessing the highest survival, activation and migratory capacity (63). Birjandi et al. suggested that regulatory cells lose their suppressor function and polarize toward the Th2 phenotype, promoting fibrosis progression (65). Our results indicate that similar alterations could also be expected in stable, treatment-receiving IPF patients. The frequent presence of IL-17-producing Tregs has been recognized in various inflammatory diseases or their animal models (66), but, to the best of our knowledge, this is the first report of Th-17-like Tregs in IPF. These Th-2- and Th-17-like Tregs may contribute to disease progression.

Analyses of ICOS+PD-1+ Tregs revealed reduced proportions of these cells in IPF. The decrease in of this population may suggest compromised functionality of Tregs in our IPF group as previously indicated by others (61). Reilkoff et al. recognized increased expression of the functional deficiency marker semaphorin 7a in Tregs from progressive IPF patients (67). However, our publication is the very first to evaluate the expression of ICOS and PD-1 on Tregs in stable IPF.

Th1 effectors are generally recognized for their powerful antifibrotic effects via, inter alia, IFN-γ or IL-12 synthesis (68). T-bet-deficient mice are known to be more susceptible to bleomycin-induced fibrosis (69). It is believed that the Th1/Th2 balance within lung tissue may define whether injury will be resolved or fibrosis will develop (6). Lung fibrosis is often recognized as Th-2-based disorder with a Th2 cytokines promoting: fibroblasts activation and proliferation, FMT, ECM production and collagen deposition (70–72).

After vaccine administration, the proportions of effector T lymphocytes were reduced in our IPF group. Naïve Teffs were decreased whereas memory cells were increased in IPF when compared to control group at all established time points. A decline in the proportion of Teffs has already been reported in nintedanib-treated patients (59).

The proportions of general and naïve Th1 effector lymphocytes were reduced in IPF. The ratios of memory cells were also decreased in patients before vaccination but increased after the procedure. A higher proportion of general Th2 cells were characteristic of IPF. We have also observed lower ratios of naïve Th2 cells and increased proportions of memory Th2 lymphocytes in patients. The proportions of Th17 effectors and their naïve subpopulation decreased in the IPF group after vaccination, whereas the percentages of for memory Th17 cells were heightened in patients when compared to healthy volunteers. Patients also displayed reduced proportions of Th1/17 effectors, and their various subpopulations compared to control subjects. Previous publications have described similar proportions of Th1 lymphocytes in patients and healthy individuals (59, 60). To the best of our knowledge, this is the first publication to report a decreased proportions of circulating Th-1 effectors in IPF. Predominance of Th-2 responses in IPF has been recognized (73). However, contrary to our results, d’Alessandro et al. did not observe differences in the proportions of Th2 cells between treated or untreated IPF patients and healthy volunteers (59). Reduced proportions of circulating Th17 lymphocytes in newly diagnosed IPF patients have been reported (59, 60). Galati et al. reported reduced proportions of IL-17-producing CD8- T cells in IPF (58). d’Alessandro et al. also reported decreased proportions of Th1/17 cells in nintedanib-treated patients (59). Altered proportions of effector cells that we have observed could, at least partially, result from heightened ratios of Tregs in IPF as implied by animal model experiments (56, 74).

Cytotoxic T lymphocytes are considered destructive when the development and progression of IPF is concerned and as such are even appointed possible therapeutic target (6).

Our IPF patients had significantly higher proportions of Tc lymphocytes and decreased Th/Tc ratios than did the healthy group. Contradictory to our results, Galati et al. recognized similar proportions of Tc lymphocytes between IPF and healthy subjects. Moreover, differences in the Th/Tc ratio were not observed between the two groups (58). The ratio of CD4/CD8<1 has been connected with immune senescence or persistent viral infections. The CD4/CD8 ratios are reduced in patients with progressive IPF rather than in stable sufferers (60). Subpopulations of Tc cells that were heightened in our patients included recent thymic emigrants as well as central and effector memory cells. Increase in naïve as well as central memory Tc cells in stable IPF patients have been reported (60). CD28 is a major costimulatory molecule required for full T cell activation (75). Downregulated expression of the receptor on the PBMCs of IPF patients has been described (58) and recognized as an independent poor prognostic factor (76). Reduced CD28 expression also implies constant proliferation followed by clonal exhaustion (77). Patients with decreased expression levels of CD28 on PBMCs are characterized by reduced transplant-free survival (78). CD28+ Tc were heightened in the IPF group before vaccination. However, patients were also characterized by increased proportions of senescent CD28-CD57+ Tc lymphocytes and their CM and EM subpopulations at all time points. Mendoza et al. also described increased proportions of exhausted cytotoxic T cells, distinguished as CD28-CD8+ cells in newly diagnosed IPF (60). It has been documented that the percentages of CD8+ cells as well as their effector memory and CD28- subpopulations are lower in stable IPF patients when compared to those with progressive IPF. The ratios of CD28− Tc cells also correlate with decline in FVC and DLCO parameters (60). Our IPF group also had reduced ratios of FasL+ cytotoxic T cells than the control group. Kopiński et al. reported elevated FasL+ expression on Tc cells in BALF samples of IPF patients (79), but the analyses of ligand expression n peripheral blood circulating Tc have not yet been reported.

## 5 Conclusion

This study was conducted to investigate immune parameters in IPF patients compared to healthy individuals, focusing on their response to the BNT162b2 vaccine. The primary goal was to assess the immunoglobulin production post-vaccination, particularly IgG, crucial for long-term immunity. Despite similar IgG seroconversion rates, IPF patients displayed lower antibody concentrations after the first vaccination, possibly due to ongoing disease or antifibrotic therapy. This reduced response was consistent after the second dose and observed by other researchers as well.

Th-1 responses, important for protection against SARS-CoV-2, were compromised in IPF patients post-vaccination, as indicated by reduced IFNγ secretion. Elevated IL-6 and IL-22 levels in IPF patients suggest ongoing inflammation and fibrotic processes, potentially impacting vaccine responses.

Analysis of T cell populations revealed heightened proportions of regulatory T cells in IPF patients, particularly those with Th2 and Th17-like phenotypes, which may contribute to disease progression. Reduced Th1 responses and altered Th17 proportions were observed post-vaccination, indicating dysregulation of adaptive immunity. Cytotoxic T lymphocytes were elevated in IPF patients, along with increased proportions of senescent Tc cells, suggesting immune senescence and potential implications for disease progression.

Idiopathic pulmonary fibrosis, with its progression resulting from aberrant immune responses, is sometimes compared to cancer rather than an inflammatory disorder. It is still unclear why IPF progresses in some treatment-receiving patients. Our results indicate that numerous aberrations in adaptive immune responses can be distinguished in the circulating lymphocytes of IPF patients despite the use of antifibrotic therapies. These results imply dysregulation and exhaustion of both helper and cytotoxic lymphocytes even in treated patients. Such immune senescence should be recognized as a subject of new studied and new therapeutic targets.

Overall, the study highlights immune dysregulation in IPF patients. These findings underscore the need for tailored vaccination strategies and further research to address immune dysfunction in IPF, particularly in the context of emerging infectious diseases such as COVID-19. Understanding immune dynamics in IPF is essential for optimizing patient care and therapeutic interventions.

## Data Availability

The raw data supporting the conclusions of this article will be made available by the authors, without undue reservation.
