# Supplementary material for: Comprehensive analyses of immune activity in COVID-19-vaccinated idiopathic pulmonary fibrosis patients
Source: Front Immunol. 2025 Jan 8;15:1436491. doi: 10.3389/fimmu.2024.1436491 (PMC11750670; doi:10.3389/fimmu.2024.1436491)
Supplement: Supplementary file 1 [file DataSheet1.docx]

Supplementary Material

**Comprehensive analyses of immune activity in COVID-19-vaccinated
Idiopathic Pulmonary Fibrosis patients**

**Table 1.** Antibodies used in cellular staining for flow cytometry analysis (antibody/manufacturer/catalogue identification). The antibodies, categorized into 3 distinct types of staining, were applied to distinguish, and identify various cellular populations*.*

| Unstained | IRP | Tregs | B Phenotype |
| --- | --- | --- | --- |
| CD45  [eBioscience](https://www.googleadservices.com/pagead/aclk?sa=L&ai=DChcSEwjEyfSJjPH4AhV1RZEFHYuFA-4YABAAGgJscg&ae=2&ohost=www.google.com&cid=CAESbeD2gwn8XDtD13C101RSvP9Lebjztw57dWCpbuvOZ4OHyFuPVLbPE9J6U5jwpc0-5vqVZgZDAYQ7sznnnNVTwjr0uUESWFAWRwmFHAiUFopC4gk-cMy70dy2iIDPvlsQCh73r0BkaDoJdaiV_5M&sig=AOD64_09XsW5XTqDXrvFjoZ-3PDQW_rzcA&q&adurl&ved=2ahUKEwioju6JjPH4AhXIUXcKHR1lCP8Q0Qx6BAgEEAE)  #MHCD4530 | CD45  eBioscience #MHCD4530 | CD45  eBioscience #MHCD4530 | CD45  eBioscience #MHCD4530 |
| CD3  eBioscience  #11-0037-42 | CD3  eBioscience  #11-0037-42 | CD3  eBioscience  #11-0037-42 | CD3  eBioscience  #11-0037-42 |
| CD4  eBioscience  #MA1-19775 | CD4  eBioscience  #MA1-19775 | CD4  eBioscience  #MA1-19775 | - |
| CD8  BD  #563795 | CD8  BD  #563795 | - | IgM  BD  #563903 |
| - | CD56  BD  #612766 | CD127  BD  #612794 | - |
| - | CD16  eBioscience  #78-0168-42 | HLA-DR  eBioscience  #78-9956-42 | CD5  eBioscience  #78-0059-42 |
| Zombie Red  Biolegend  #423110 | Zombie Red  Biolegend  #423110 | Zombie Red  Biolegend  #423110 | Zombie Red  Biolegend  #423110 |
| - | CD197 (CCR7)  eBioscience  #47-1979-42 | CD197 (CCR7)  eBioscience  #47-1979-42 | CD24  eBioscience  #47-0247-42 |
| - | CD45RA  eBioscience  #63-0458-42 | CD45RA  eBioscience  #63-0458-42 | CD10  eBioscience  #63-0106-42 |
| - | TCRγδ  Biolegend  #331212 | CD196 (CCR6)  eBioscience  #17-1969-42 | CD21  eBioscience  #17-0219-42 |
| - | CD28  eBioscience  #56-0289-42 | CD278 (ICOS)  eBioscience  #56-9948-42 | CD38  eBioscience  #56-0389-42 |
| - | CD178 (FasL)  eBioscience  #12-9919-42 | CD183 (CXCR3)  eBioscience  #12-1839-42 | IgD  eBioscience  #12-9868-42 |
| - | CD31  eBioscience  #25-0319-42 | CD25  eBioscience  #25-0257-42 | CD19  eBioscience  #25-0199-42 |
| - | CD57  eBioscience  #48-0577-42 | CD45RO  eBioscience  #48-0457-42 | - |
| - | CD27  eBioscience  #64-0279-42 | CD185 (CXCR5)  eBioscience  #64-9185-42 | CD27  eBioscience  #64-0279-42 |
| - | TCR Vα7.2  Biolegend  #351732 | CD279 (PD1)  eBioscience  #67-2799-42 | - |

**Figure 1.** Gating strategy

1. Gating strategy of selected example populations of T helper, cytotoxic, regulatory and effector cells.


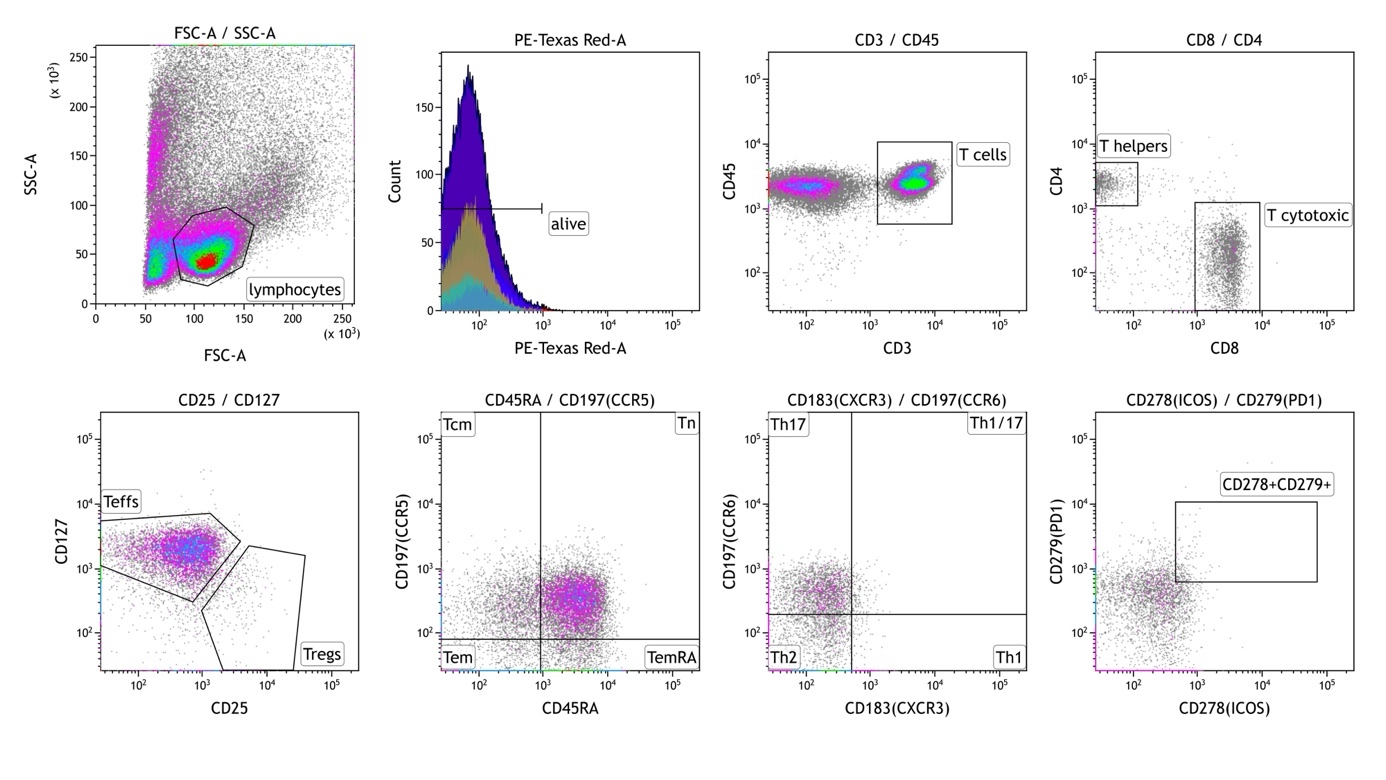

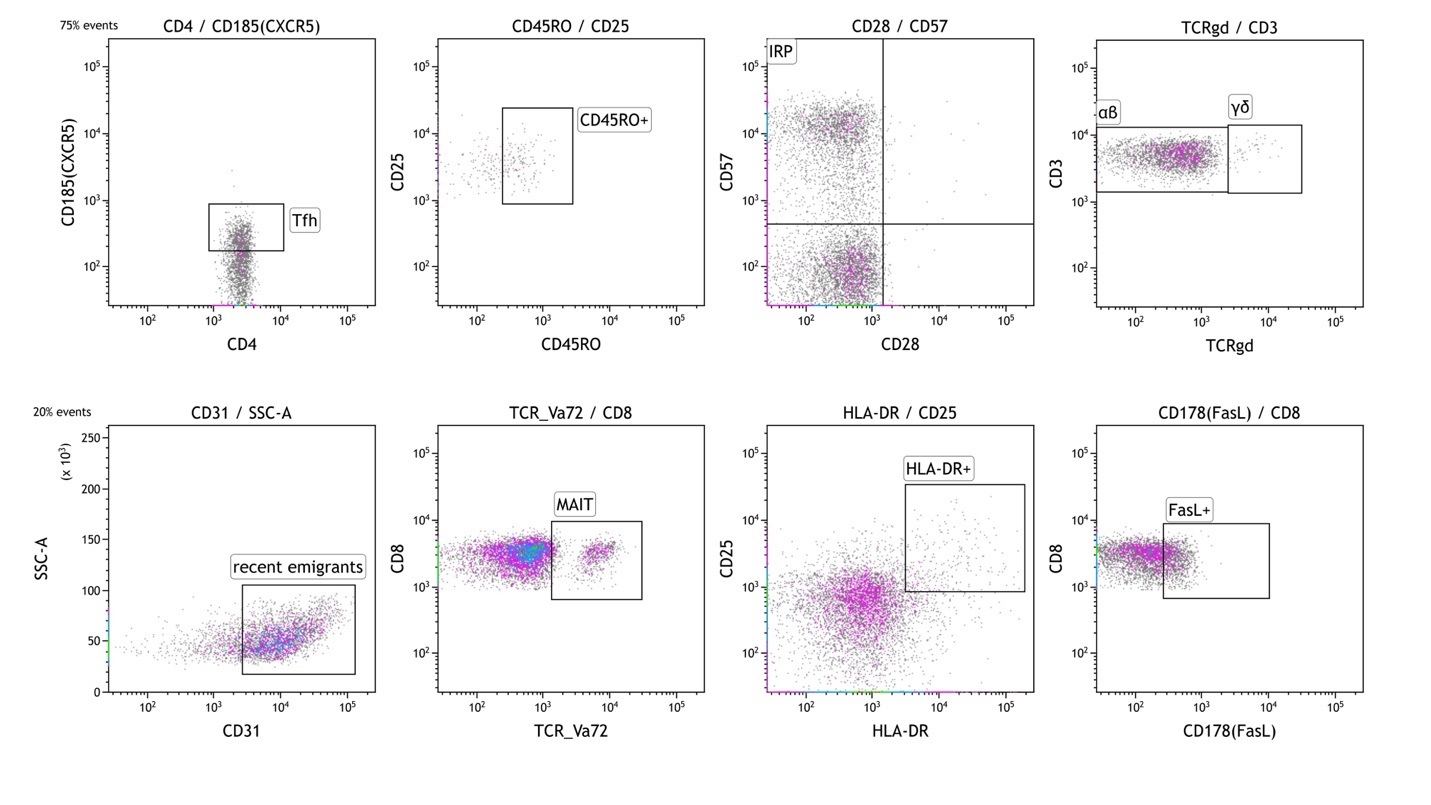


1. Gating strategy of B lymphocytes and subpopulations of CD19+ cells.


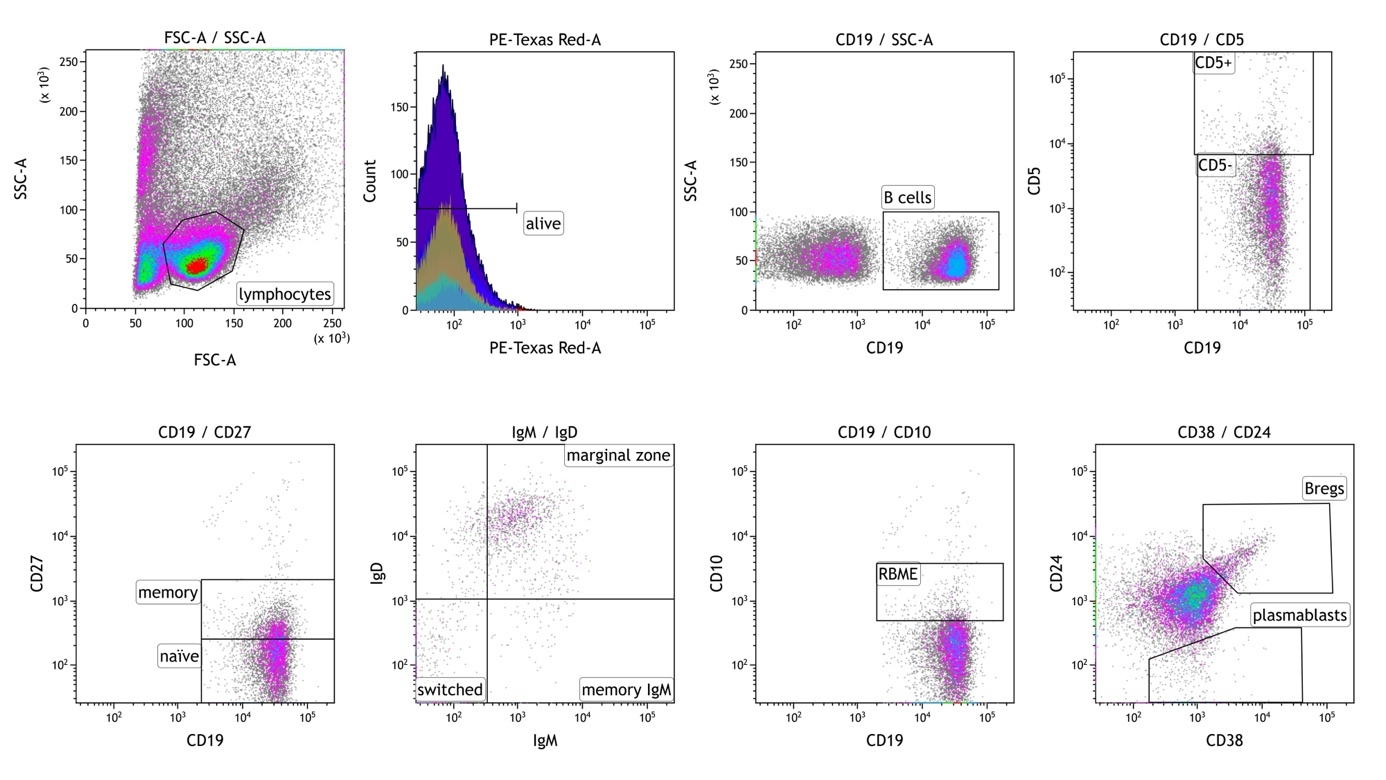

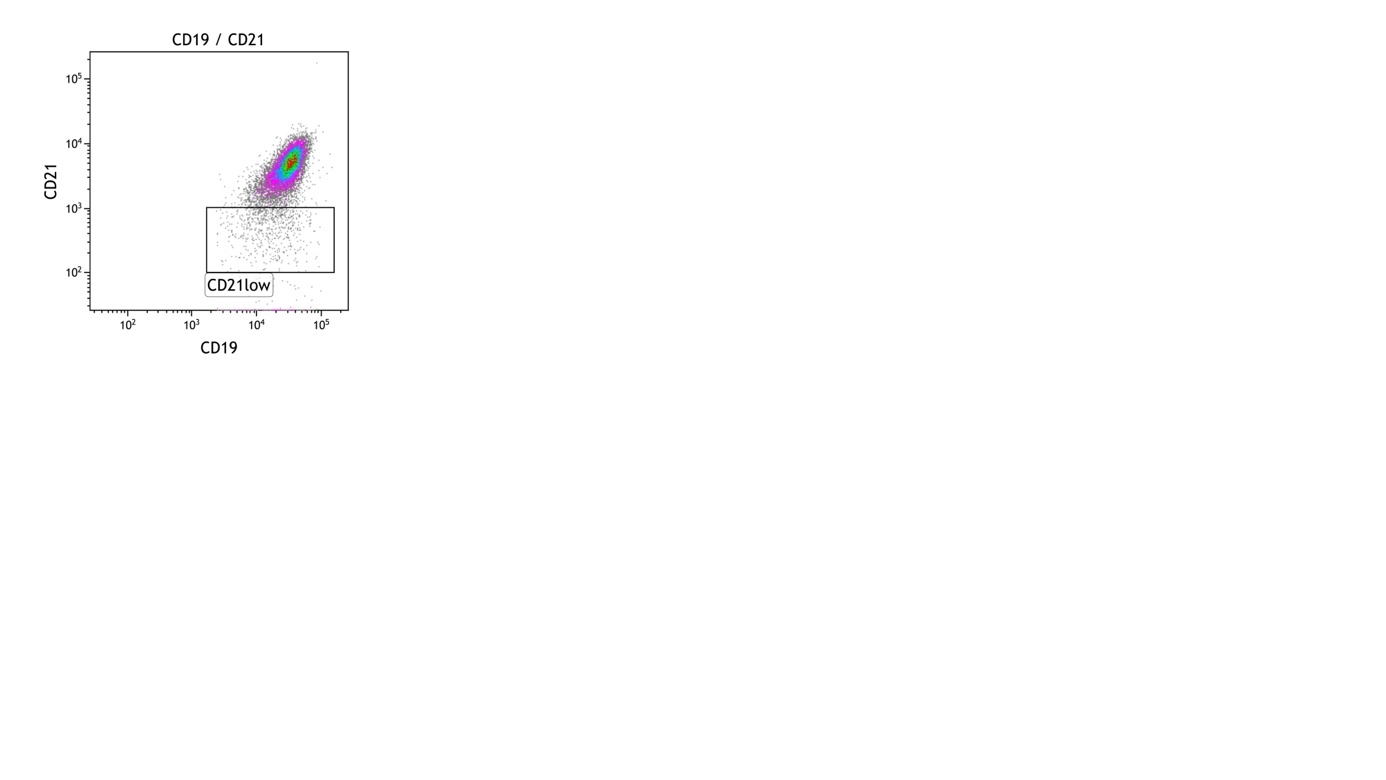


1. Gating strategy of NK cells and their subpopulations.


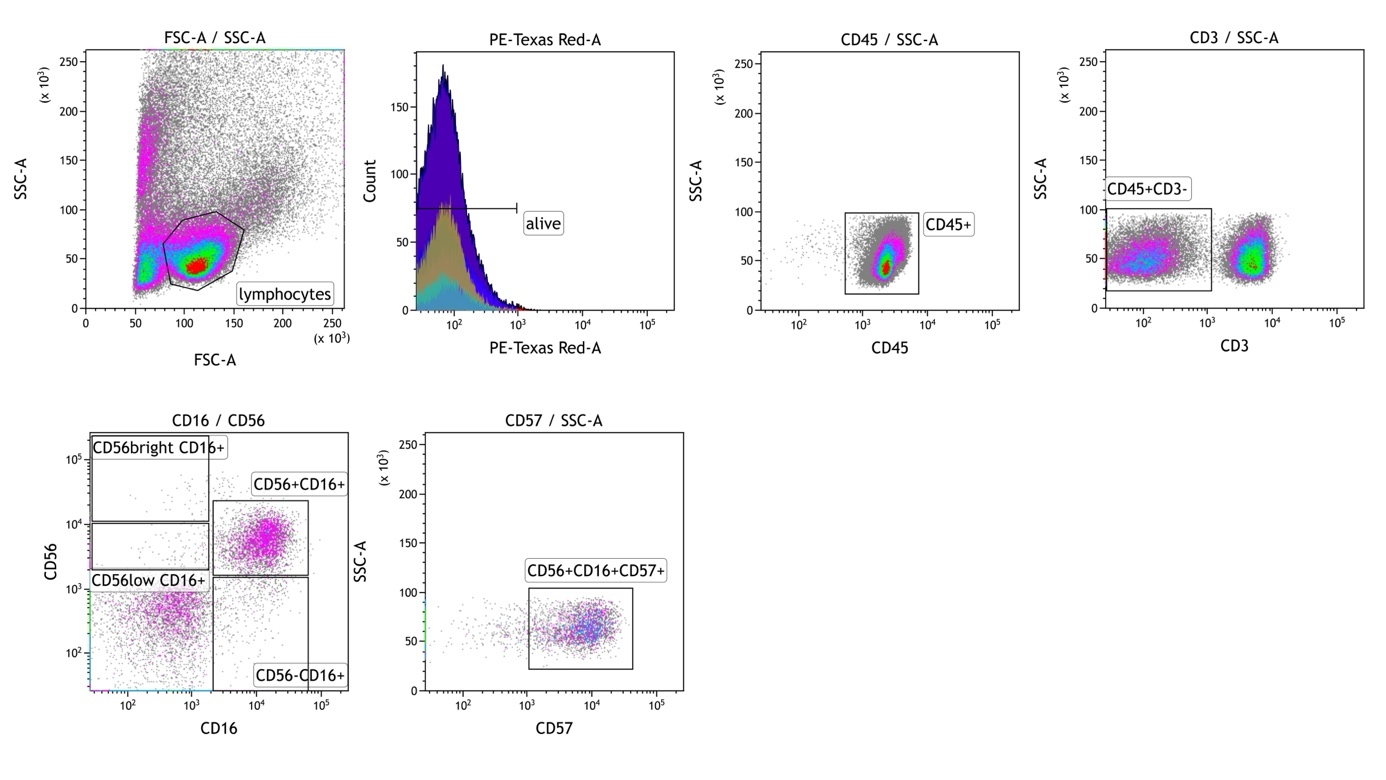


**Table 2.** The analyzed populations, which exhibited significant differences between patients and control group, along with their phenotypes.

| **Group** | **Analyzed population** | **Phenotype** |
| --- | --- | --- |
| T cells | T regulatory cells | CD45+CD3+CD4+CD25+ |
|  | T effector cells | CD45+CD3+CD4+CD127+ |
| T regulatory cells | Th1 Tregs | CD45+CD3+CD4+CD25+CD196-CD183+ |
|  | Th2 Tregs | CD45+CD3+CD4+CD25+CD196-CD183- |
|  | Th17 Tregs | CD45+CD3+CD4+CD25+CD196+CD183- |
|  | Th1/17 Tregs | CD45+CD3+CD4+CD25+CD196+CD183+ |
|  | ICOS+PD-1+Tregs | CD45+CD3+CD4+CD25+CD278+CD279+ |
|  | Follicular Tregs | CD45+CD3+CD4+CD25+CD278+CD279+CD185+ |
|  | Naïve Tregs | CD45+CD3+CD4+CD25+CD197+CD45RA+ |
|  | CM Tregs | CD45+CD3+CD4+CD25+CD197+CD45RA- |
|  | EM Tregs | CD45+CD3+CD4+CD25+CD197-CD45RA- |
|  | Th1 CM Tregs | CD45+CD3+CD4+CD25+CD196-CD183+CD197+CD45RA- |
|  | Th1 EM Tregs | CD45+CD3+CD4+CD25+CD196-CD183+CD197-CD45RA- |
|  | Th2 CM Tregs | CD45+CD3+CD4+CD25+CD196-CD183-CD197+CD45RA- |
|  | Th2 EM Tregs | CD45+CD3+CD4+CD25+CD196-CD183-CD197-CD45RA- |
|  | Th17 CM Tregs | CD45+CD3+CD4+CD25+CD196+CD183-CD197+CD45RA- |
|  | Th17 EM Tregs | CD45+CD3+CD4+CD25+CD196+CD183-CD197-CD45RA- |
|  | Th1/17 CM Tregs | CD45+CD3+CD4+CD25+CD196+CD183+CD197+CD45RA- |
|  | Th1/17 EM Tregs | CD45+CD3+CD4+CD25+CD196+CD183+CD197-CD45RA- |
|  | ICOS+PD-1+naïve Tregs | CD45+CD3+CD4+CD25+CD278+CD279+CD197+CD45RA- |
|  | ICOS+PD-1+CM Tregs | CD45+CD3+CD4+CD25+CD278+CD279+CD197-CD45RA- |
|  | ICOS+PD-1+EM Tregs | CD45+CD3+CD4+CD25+CD278+CD279+CD197-CD45RA+ |
|  | Follicular naïve Tregs | CD45+CD3+CD4+CD25+CD278+CD279+CD185+CD197+CD45RA- |
|  | Follicular CM Tregs | CD45+CD3+CD4+CD25+CD278+CD279+CD185+CD197-CD45RA- |
|  | Follicular EM Tregs | CD45+CD3+CD4+CD25+CD278+CD279+CD185+CD197-CD45RA+ |
| T effector cells | Th1 Teffs | CD45+CD3+CD4+CD127+CD196-CD183- |
|  | Th2 Teffs | CD45+CD3+CD4+CD127+CD196+CD183- |
|  | Th17 Teffs | CD45+CD3+CD4+CD127+CD196+CD183+ |
|  | Th1/17 Teffs | CD45+CD3+CD4+CD127+CD278+CD279+ |
|  | Follicular Teffs | CD45+CD3+CD4+CD127+CD197+CD45RA+ |
|  | Naïve Teffs | CD45+CD3+CD4+CD127+CD197+CD45RA- |
|  | CM Teffs | CD45+CD3+CD4+CD127+CD197-CD45RA- |
|  | EM Teffs | CD45+CD3+CD4+CD127+CD197-CD45RA+ |
|  | Th1 CM Teffs | CD45+CD3+CD4+CD127+CD196-CD183+CD197-CD45RA- |
|  | Th1 EM Teffs | CD45+CD3+CD4+CD127+CD196-CD183+CD197-CD45RA+ |
|  | Th2 CM Teffs | CD45+CD3+CD4+CD127+CD196-CD183-CD197-CD45RA+ |
|  | Th2 EM Teffs | CD45+CD3+CD4+CD127+CD196+CD183-CD197+CD45RA+ |
|  | Th17 CM Teffs | CD45+CD3+CD4+CD127+CD196+CD183-CD197-CD45RA- |
|  | Th17 EM Teffs | CD45+CD3+CD4+CD127+CD196+CD183-CD197-CD45RA+ |
|  | Th1/17 CM Teffs | CD45+CD3+CD4+CD127+CD196+CD183+CD197+CD45RA- |
|  | Th1/17 EM Teffs | CD45+CD3+CD4+CD127+CD196+CD183+CD197-CD45RA- |
|  | Follicular naïve Teffs | CD45+CD3+CD4+CD127+CD278+CD279+CD185+CD197+CD45RA+ |
|  | Follicular CM Teffs | CD45+CD3+CD4+CD127+CD278+CD279+CD185+CD197+CD45RA- |
|  | Follicular EM Teffs | CD45+CD3+CD4+CD127+CD278+CD279+CD185+CD197-CD45RA- |
| T cells | T helpers | CD45+CD3+CD4+ |
|  | T cytotoxic | CD45+CD3+CD8+ |
| T helper cells | Recent emigrants Th | CD45+CD3+CD4+ CD197+CD45RA+ CD31+ |
|  | Naïve Th | CD45+CD3+CD4+CD197+CD45RA+ |
|  | CM Th | CD45+CD3+CD4+CD197+CD45RA- |
|  | EM Th | CD45+CD3+CD4+CD197-CD45RA- |
|  | IRP Th | CD45+CD3+CD4+CD57+CD28- |
|  | IRP CM Th | CD45+CD3+CD4+CD57+CD28-CD197+CD45RA- |
|  | IRP EM Th | CD45+CD3+CD4+CD57+CD28-CD197-CD45RA- |
|  | MAIT Th | CD45+CD3+CD4+TCRVa7.2+ |
|  | Recent emigrants MAIT Th | CD45+CD3+CD4+TCRVa7.2+ CD197+CD45RA+ CD31+ |
|  | MAIT naïve Th | CD45+CD3+CD4+TCRVa7.2+CD197+CD45RA+ |
|  | MAIT CM Th | CD45+CD3+CD4+TCRVa7.2+CD197+CD45RA- |
|  | MAIT EM Th | CD45+CD3+CD4+TCRVa7.2+CD197-CD45RA- |
|  | IRP MAIT Th | CD45+CD3+CD4+TCRVa7.2+ CD57+CD28- |
|  | IRP MAIT CM Th | CD45+CD3+CD4+TCRVa7.2+CD197+CD45RA- CD57+CD28- |
|  | IRP MAIT EM Th | CD45+CD3+CD4+TCRVa7.2+CD197-CD45RA- CD57+CD28- |
|  | $\gamma\delta$ Th | CD45+CD3+CD4+TCRgd+ |
|  | Recent emigrants $\gamma\delta$Th | CD45+CD3+CD4+TCRgd+ CD197+CD45RA+CD31+ |
|  | $\gamma\delta$naïve Th | CD45+CD3+CD4+TCRgd+CD197+CD45RA+ |
|  | $\gamma\delta$ CM Th | CD45+CD3+CD4+TCRgd+CD197+CD45RA- |
|  | $\gamma\delta$ EM Th | CD45+CD3+CD4+TCRgd+CD197-CD45RA- |
|  | $\gamma\delta$ IRP | CD45+CD3+CD4+TCRgd+CD57+CD28- |
|  | $\gamma\delta$ IRP CM Th | CD45+CD3+CD4+TCRgd+CD57+CD28-CD197+CD45RA- |
|  | $\gamma\delta$ IRP EM Th | CD45+CD3+CD4+TCRgd+CD57+CD28-CD197-CD45RA- |
|  | CD27+ Th | CD45+CD3+CD4+CD27+ |
|  | Naïve CD27+ Th | CD45+CD3+CD4+CD197+CD45RA+CD27+ |
|  | CM CD27+ Th | CD45+CD3+CD4+CD197+CD45RA- CD27+ |
|  | EM CD27+ Th | CD45+CD3+CD4+CD197-CD45RA- CD27+ |
|  | CD27- Th | CD45+CD3+CD4+CD27- |
|  | CD28+ Th | CD45+CD3+CD4+CD28+ |
|  | CD57+ Th | CD45+CD3+CD4+CD57+ |
|  | FasL+ Th | CD45+CD3+CD4+FasL+ |
| T cytotoxic cells | Recent emigrants Tc | CD45+CD3+CD8+ CD197+CD45RA+ CD31+ |
|  | Naïve Tc | CD45+CD3+CD8+CD197+CD45RA+ |
|  | CM Tc | CD45+CD3+CD8+CD197+CD45RA- |
|  | EM Tc | CD45+CD3+CD8+CD197-CD45RA- |
|  | TEMRA Tc | CD45+CD3+CD8+CD197-CD45RA+ |
|  | IRP Tc | CD45+CD3+CD8+CD57+CD28- |
|  | IRP CM Tc | CD45+CD3+CD8+CD57+CD28-CD197+CD45RA- |
|  | IRP EM Tc | CD45+CD3+CD8+CD57+CD28-CD197-CD45RA- |
|  | IRP TEMRA Tc | CD45+CD3+CD8+CD57+CD28-CD197-CD45RA+ |
|  | MAIT Tc | CD45+CD3+CD8+TCRVa7.2+ |
|  | Recent emigrants MAIT Tc | CD45+CD3+CD8+TCRVa7.2+ CD197+CD45RA+ CD31+ |
|  | MAIT naïve Tc | CD45+CD3+CD8+TCRVa7.2+CD197+CD45RA+ |
|  | MAIT CM Tc | CD45+CD3+CD8+TCRVa7.2+CD197+CD45RA- |
|  | MAIT EM Tc | CD45+CD3+CD8+TCRVa7.2+CD197-CD45RA- |
|  | MAIT TEMRA Tc | CD45+CD3+CD8+TCRVa7.2+CD197-CD45RA+ |
|  | IRP MAIT naïve Tc | CD45+CD3+CD8+TCRVa7.2+CD197+CD45RA+ CD57+CD28- |
|  | IRP MAIT CM Tc | CD45+CD3+CD8+TCRVa7.2+CD197+CD45RA- CD57+CD28- |
|  | IRP MAIT EM Tc | CD45+CD3+CD8+TCRVa7.2+CD197-CD45RA- CD57+CD28- |
|  | IRP MAIT TEMRA Tc | CD45+CD3+CD8+TCRVa7.2+CD197-CD45RA+ CD57+CD28- |
|  | $\gamma\delta$ Tc | CD45+CD3+CD8+TCRgd+ |
|  | Recent emigrants $\gamma\delta$ Tc | CD45+CD3+CD8+TCRgd+ CD197+CD45RA+CD31+ |
|  | $\gamma\delta$naïve Tc | CD45+CD3+CD8+TCRgd+CD197+CD45RA+ |
|  | $\gamma\delta$ CM Tc | CD45+CD3+CD8+TCRgd+CD197+CD45RA- |
|  | $\gamma\delta$ EM Tc | CD45+CD3+CD8+TCRgd+CD197-CD45RA- |
|  | $\gamma\delta$TEMRA Tc | CD45+CD3+CD8+TCRgd+CD197-CD45RA+ |
|  | $\gamma\delta$ IRP Tc | CD45+CD3+CD8+TCRgd+CD57+CD28- |
|  | $\gamma\delta$ IRP CM Tc | CD45+CD3+CD8+TCRgd+CD57+CD28-CD197+CD45RA- |
|  | $\gamma\delta$ IRP EM Tc | CD45+CD3+CD8+TCRgd+CD57+CD28-CD197-CD45RA- |
|  | $\gamma\delta$ IRP TEMRA Tc | CD45+CD3+CD8+TCRgd+CD57+CD28-CD197-CD45RA+ |
|  | CD27+ Tc | CD45+CD3+CD8+CD27+ |
|  | Naïve CD27+ Tc | CD45+CD3+CD8+CD197+CD45RA+CD27+ |
|  | CM CD27+ Tc | CD45+CD3+CD8+CD197+CD45RA- CD27+ |
|  | EM CD27+ Tc | CD45+CD3+CD8+CD197-CD45RA- CD27+ |
|  | TEMRA CD27+ Tc | CD45+CD3+CD8+CD197-CD45RA+ CD27+ |
|  | CD27- Tc | CD45+CD3+CD8+CD27- |
|  | CD28+ Tc | CD45+CD3+CD8+CD28+ |
|  | CD57+ Tc | CD45+CD3+CD8+CD57+ |
|  | FasL+ Tc | CD45+CD3+CD8+FasL+ |
| NK cells | CD3-CD56brightCD16- | CD3-CD56brightCD16- |
|  | CD3-CD56+CD16+ | CD3-CD56+CD16+ |
|  | CD3-CD56+CD16+CD57+ | CD3-CD56+CD16+CD57+ |
|  | CD3-CD56-CD16+ | CD3-CD56-CD16+ |
|  | CD3-CD56lowCD16- | CD3-CD56lowCD16- |
| B cells | B cells | CD19+CD20+ |
|  | CD5+ | CD19+CD20+CD5+ |
|  | CD5- | CD19+CD20+CD5- |
|  | RBME | CD19+CD20+CD10+ |
|  | Memory B | CD19+CD20+CD27+ |
|  | Marginal zone | CD19+CD20+CD27+IgM+IgD+ |
|  | Switched B | CD19+CD20+CD27+IgM-IgD- |
|  | Memory IgM | CD19+CD20+CD27+IgM+IgD- |
|  | Naive B | CD19+CD20+CD27- |
|  | Bregs | CD19+CD20+CD38+CD24+ |
|  | Plasmablasts | CD19+CD20+CD38+CD24- |
|  | CD21low | CD19+CD20+CD21low |

**Table 3.** The clinical characteristic of patients included into the study. The data are presented as medians (IQRs).

| Analysis | Parameter | IPF n=17 | IPF conv. n=7 |
| --- | --- | --- | --- |
| Spirometry | FEV1 (L) | 2.33 (1.92-2.43) | 2.16 (1.92-2.89) |
|  | FVC (L) | 2.66 (2.39-3.04) | 2.56 (2.33-3.19) |
|  | FVC IN (L) | 2.74 (2.45-3.16) | 2.29 (2.48-3.17) |
|  | FEV 1 % FVC (%) | 81.56 (79.13-86.57) | 83.44 (81.44-88.62) |
|  | FEV1 % VC MAX (%) | 78.41 (73.27-84.62) | 80.84 (77.49-84.59) |
|  | PEF (L/s) | 7.93 (7.29-9.59) | 7.27 (6.76-9.82) |
|  | MEF 75 (L/s) | 7.72 (5.74-8.61) | 7.18 (6.38-8.65) |
|  | MEF 50 (L/s) | 3.53 (2.11-5.07) | 3.28 (2.80-4.21) |
|  | MEF 25 (L/s) | 0.55 (0.42-0.84) | 0.63 (0.51-0.75) |
| Biochemistry | Total bilirubin (mg/dL) | 0.69 (0.56-0.96) | 0.62 (0.44-0.78) |
|  | Creatinine (mg/dL) | 0.97 (0.75-1.35) | 0.75 (0.71-0.78) |
|  | eGFR (mL/min/1.73m) | 78.00 (51.00-90.00) | 90.00 (90.00-90.00) |
| Complete blood count | RBC (x10^12^/L) | 4.69 (4.39-5.01) | 4.69 (4.11-5.15) |
|  | Hemoglobin (g/dL) | 14.45 (13.80-15.45) | 14.00 (13.80-16.00) |
|  | PLT (x10^9^/L) | 227.50 (202.00-289.50) | 183.00 (142.00-246.00) |
|  | WBC (x10^9^/L) | 8.70 (7.95-10.34) | 8.40 (6.25-8.97) |
|  | % Neutrophils (%) | 64.40 (60.45-69.35) | 57.40 (55.60-58.60) |
|  | % Lymphocytes (%) | 22.00 (18.75-27.80) | 32.70 (29.40-33.60) |
|  | % Monocytes (%) | 7.55 (6.85-8.55) | 6.70 (6.50-9.30) |
|  | % Eosinophils (%) | 3.25 (2.20-4.45) | 2.50 (1.80-2.70) |
|  | % Basophils (%) | 0.70 (0.55-0.75) | 0.60 (0.50-0.90) |

**Table 4.** Altered differentiation of regulatory T lymphocytes in IPF

| Population | before | | | 1^st^ | | | 2^nd^ | | |
| --- | --- | --- | --- | --- | --- | --- | --- | --- | --- |
|  | Median (%) | | *P* value | Median (%) | | *P* value | Median (%) | | *P* value |
|  | IPF | control |  | IPF | control |  | IPF | control |  |
| T regulatory cells | 10.51 | 7.78 | **0.002** | 14.46 | 9.23 | **0.006** | 16.40 | 8.65 | **<0.000** |
| Naïve Tregs | 1.61 | 2.19 | 0.91 | 1.97 | 2.11 | 0.60 | 3.60 | 2.34 | 0.08 |
| CM Tregs | 2.47 | 2.40 | 0.48 | 2.72 | 2.01 | **0.01** | 3.48 | 1.84 | **<0.000** |
| EM Tregs | 3.43 | 2.08 | **0.001** | 4.66 | 2.21 | **<0.000** | 4.09 | 2.02 | **<0.000** |

**Table 5.** Effect of IPF on Tregs phenotype during differentiation.

| Population | before | | | 1^st^ | | | 2^nd^ | | |
| --- | --- | --- | --- | --- | --- | --- | --- | --- | --- |
|  | Median (%) | | *P* value | Median (%) | | *P* value | Median (%) | | *P* value |
|  | IPF | control |  | IPF | control |  | IPF | control |  |
| Th1 Tregs | 1.39 | 2.35 | **0.01** | 3.41 | 2.78 | 0.55 | 3.99 | 2.10 | **0.008** |
| Th2 Tregs | 4.56 | 1.79 | **<0.000** | 4.87 | 2.49 | **<0.000** | 5.34 | 2.20 | **<0.000** |
| Th17 Tregs | 3.68 | 1.64 | **<0.000** | 3.99 | 1.93 | **<0.000** | 3.22 | 2.02 | **<0.000** |
| Th1/17 Tregs | 1.39 | 2.20 | **<0.000** | 2.36 | 2.59 | 0.83 | 2.90 | 2.21 | 0.20 |
| Th1 CM Tregs | 0.21 | 0.68 | **0.001** | 0.66 | 0.57 | 0.63 | 0.99 | 0.49 | **0.004** |
| Th1 EM Tregs | 0.34 | 0.55 | **0.03** | 0.62 | 0.53 | 0.74 | 0.91 | 0.46 | **0.001** |
| Th2 CM Tregs | 1.36 | 0.75 | **0.004** | 1.13 | 0.47 | **0.004** | 1.39 | 0.56 | **<0.000** |
| Th2 EM Tregs | 1.70 | 0.54 | **<0.000** | 1.99 | 0.65 | **<0.000** | 1.40 | 0.53 | **<0.000** |
| Th17 CM Tregs | 0.80 | 0.42 | **0.04** | 0.67 | 0.30 | 0.07 | 0.55 | 0.28 | **0.003** |
| Th17 EM Tregs | 1.44 | 0.43 | **<0.000** | 1.29 | 0.48 | **<0.000** | 0.88 | 0.42 | **<0.000** |
| Th1/17 CM Tregs | 0.30 | 0.49 | **0.01** | 0.67 | 0.27 | 0.83 | 0.77 | 0.43 | **0.03** |
| Th1/17 EM Tregs | 0.13 | 0.37 | **0.001** | 0.61 | 0.34 | 0.06 | 0.44 | 0.31 | **0.05** |
| ICOS+PD-1+Tregs | 0.33 | 0.64 | **0.02** | 0.81 | 0.45 | 0.40 | 1.09 | 0.74 | 0.27 |
| ICOS+PD-1+naïve Tregs | 0.05 | 0.22 | 0.46 | 0.12 | 0.12 | 0.74 | 0.24 | 0.18 | 0.40 |
| ICOS+PD-1+CM Tregs | 0.06 | 0.08 | 0.91 | 0.08 | 0.07 | 0.22 | 0.10 | 0.08 | 0.17 |
| ICOS+PD-1+EM Tregs | 0.15 | 0.17 | 0.50 | 0.31 | 0.17 | 0.12 | 0.38 | 0.22 | 0.07 |
| Follicular Tregs | 0.02 | 0.01 | 0.58 | 0.04 | 0.00 | 0.20 | 0.06 | 0.06 | 0.29 |
| Follicular naïve Tregs | 0.00 | 0.00 | 0.71 | 0.00 | 0.00 | 0.23 | 0.01 | 0.00 | 0.17 |
| Follicular CM Tregs | 0.00 | 0.00 | 0.71 | 0.00 | 0.00 | 0.47 | 0.00 | 0.00 | 0.68 |
| Follicular EM Tregs | 0.01 | 0.00 | 0.46 | 0.02 | 0.00 | 0.07 | 0.04 | 0.01 | 0.07 |

**Table 6.** Impact of IPF on effector T lymphocytes maturation.

| Population | before | | | 1^st^ | | | 2^nd^ | | |
| --- | --- | --- | --- | --- | --- | --- | --- | --- | --- |
|  | Median (%) | | *P* value | Median (%) | | *P* value | Median (%) | | *P* value |
|  | IPF | control |  | IPF | control |  | IPF | control |  |
| T effector cells | 70.47 | 71.06 | 0.77 | 59.19 | 71.58 | **0.02** | 58.51 | 71.06 | **0.001** |
| Tregs/Teffs ratio | 0.16 | 0.11 | **0.007** | 0.24 | 0.13 | **0.007** | 0.28 | 0.12 | **<0.000** |
| Naïve Teffs | 15.59 | 25.04 | **0.04** | 11.03 | 25.85 | **0.001** | 14.36 | 30.45 | **0.001** |
| CM Teffs | 17.28 | 17.59 | 0.41 | 15.68 | 12.98 | 0.25 | 17.68 | 13.06 | **0.03** |
| EM Teffs | 23.87 | 2.67 | **0.04** | 19.63 | 12.07 | **0.02** | 15.00 | 10.69 | **0.05** |

**Table 7.** Imbalance of Teffs subpopulations in IPF. Different response to vaccination in IPF.

| Population | before | | | 1^st^ | | | 2^nd^ | | |
| --- | --- | --- | --- | --- | --- | --- | --- | --- | --- |
|  | Median (%) | | *P* value | Median (%) | | *P* value | Median (%) | | *P* value |
|  | IPF | control |  | IPF | control |  | IPF | control |  |
| Th1 Teffs | 8.15 | 26.06 | **<0.000** | 13.19 | 24.70 | **<0.000** | 19.48 | 22.95 | 0.13 |
| Th2 Teffs | 37.05 | 17.62 | **<0.000** | 23.47 | 19.83 | **0.008** | 22.89 | 19.26 | 0.31 |
| Th17 Teffs | 21.24 | 9.94 | **0.001** | 9.76 | 11.72 | 0.68 | 8.36 | 13.60 | **0.001** |
| Th1/17 Teffs | 5.22 | 17.49 | **<0.000** | 5.94 | 14.16 | **<0.000** | 7.76 | 14.76 | **<0.000** |
| Th1 CM Teffs | 1.67 | 5.12 | **<0.000** | 3.33 | 3.48 | 0.42 | 6.16 | 2.54 | **0.001** |
| Th1 EM Teffs | 1.44 | 4.43 | **0.004** | 3.49 | 2.76 | 0.42 | 4.85 | 3.00 | **0.004** |
| Th2 CM Teffs | 11.45 | 5.06 | **<0.000** | 7.84 | 5.17 | **0.01** | 7.90 | 4.98 | **0.02** |
| Th2 EM Teffs | 11.94 | 2.50 | **<0.000** | 8.08 | 3.41 | **0.001** | 4.53 | 3.07 | 0.06 |
| Th17 CM Teffs | 4.36 | 2.54 | **0.02** | 2.18 | 2.36 | 0.89 | 1.60 | 2.07 | 0.65 |
| Th17 EM Teffs | 8.36 | 1.72 | **<0.000** | 3.60 | 2.80 | 0.13 | 2.14 | 1.81 | 0.78 |
| Th1/17 CM Teffs | 0.71 | 2.87 | **<0.000** | 1.07 | 1.60 | **0.05** | 1.35 | 1.39 | 0.43 |
| Th1/17 EM Teffs | 1.38 | 3.05 | **0.004** | 1.70 | 2.07 | 0.89 | 2.22 | 1.87 | 0.27 |
| Follicular Teffs | 0.04 | 0.08 | 0.63 | 0.04 | 0.15 | **0.01** | 0.07 | 0.11 | 0.33 |
| Follicular naïve Teffs | 0.01 | 0.03 | 0.18 | 0.00 | 0.06 | **0.02** | 0.03 | 0.05 | 0.43 |
| Follicular CM Teffs | 0.03 | 0.04 | 0.91 | 0.06 | 0.13 | **0.02** | 0.05 | 0.06 | 0.95 |
| Follicular EM Teffs | 0.05 | 0.04 | 0.26 | 0.08 | 0.10 | 0.89 | 0.12 | 0.07 | 0.27 |

**Table 8.** Th cells.

| Population | before | | | 1^st^ | | | 2^nd^ | | |
| --- | --- | --- | --- | --- | --- | --- | --- | --- | --- |
|  | Median (%) | | *P* value | Median (%) | | *P* value | Median (%) | | *P* value |
|  | IPF | control |  | IPF | control |  | IPF | control |  |
| T helpers | 57.69 | 61.37 | 0.71 | 43.88 | 60.04 | 0.07 | 51.11 | 59.93 | 0.56 |
| Recent emigrants Th | 1.79 | 0.60 | **0.007** | 0.55 | 0.84 | 0.20 | 0.95 | 0.50 | 0.25 |
| Naïve Th | 11.65 | 19.66 | **0.01** | 8.83 | 19.61 | **0.001** | 13.1 | 23.95 | **0.02** |
| CM Th | 16.57 | 14.83 | 0.50 | 11.31 | 10.32 | 0.88 | 15.82 | 9.81 | 0.16 |
| EM Th | 18.98 | 9.01 | **0.006** | 14.27 | 9.76 | 0.06 | 14.03 | 9.72 | **0.02** |
| IRP Th | 2.79 | 3.23 | 0.44 | 5.01 | 2.92 | 0.43 | 2.91 | 3.06 | 0.88 |
| IRP CM Th | 0.42 | 0.46 | 0.88 | 0.74 | 0.47 | 0.40 | 0.65 | 0.35 | 0.06 |
| IRP EM Th | 1.33 | 0.59 | 0.11 | 2.21 | 0.54 | **0.01** | 1.36 | 0.63 | 0.17 |
| CD27+ Th | 17.82 | 9.12 | 0.34 | 0.64 | 2.51 | 0.29 | 0.58 | 1.37 | 0.85 |
| Naïve CD27+ Th | 6.56 | 4.65 | 0.86 | 0.41 | 1.94 | 0.14 | 0.69 | 0.81 | 0.85 |
| CM CD27+ Th | 7.71 | 3.77 | 0.35 | 0.30 | 2.39 | 0.19 | 0.77 | 1.27 | 0.78 |
| EM CD27+ Th | 2.72 | 1.94 | 0.31 | 0.39 | 1.06 | 0.45 | 0.51 | 0.63 | 0.91 |
| CD27- Th | 10.38 | 14.25 | 0.24 | 16.04 | 24.16 | 0.08 | 14.24 | 18.19 | 0.16 |
| CD28+ Th | 7.42 | 3.93 | 0.37 | 0.50 | 3.08 | **0.0006** | 0.73 | 1.73 | **0.04** |
| CD57+ Th | 3.65 | 3.39 | 1.00 | 4.23 | 3.84 | 0.98 | 3.85 | 3.37 | 0.81 |
| MAIT Th | 2.61 | 4.61 | 0.06 | 2.32 | 4.32 | **0.006** | 2.52 | 3.21 | 0.53 |
| Recent emigrant MAIT Th | 0.23 | 0.17 | 0.59 | 0.06 | 0.17 | **0.02** | 0.07 | 0.10 | 0.43 |
| MAIT naïve Th | 1.09 | 1.62 | **0.04** | 0.05 | 1.54 | **0.001** | 0.83 | 1.35 | 0.07 |
| MAIT CM Th | 0.48 | 0.80 | 0.24 | 0.63 | 0.67 | 0.40 | 0.68 | 0.35 | 0.11 |
| MAIT EM Th | 0.59 | 0.38 | 0.18 | 0.59 | 0.48 | 0.33 | 0.61 | 0.22 | 0.06 |
| IRP MAIT Th | 0.16 | 0.33 | **0.04** | 0.02 | 0.07 | 0.23 | 0.30 | 0.23 | 0.71 |
| IRP MAIT CM Th | 0.01 | 0.02 | 0.30 | 0.02 | 0.03 | 1.00 | 0.03 | 0.01 | 0.25 |
| IRP MAIT EM Th | 0.05 | 0.03 | 0.78 | 0.17 | 0.04 | 0.12 | 0.10 | 0.01 | 0.15 |
| $\boldsymbol{\gamma\delta}$ Th | 6.84 | 4.04 | **0.02** | 6.68 | 16.72 | **<0.000** | 6.74 | 1.22 | **0.007** |
| Recent emigrants $\boldsymbol{\gamma\delta}$ Th | 0.24 | 0.10 | **0.03** | 0.09 | 0.15 | 0.40 | 0.16 | 0.09 | 0.23 |
| $\boldsymbol{\gamma\delta}$naïve Th | 1.54 | 1.31 | 0.80 | 0.98 | 1.90 | 0.22 | 1.51 | 1.68 | 1.00 |
| $\boldsymbol{\gamma\delta}$ CM Th | 1.36 | 0.77 | **0.008** | 1.27 | 0.61 | **0.05** | 1.67 | 0.50 | **<0.000** |
| $\boldsymbol{\gamma\delta}$ EM Th | 2.14 | 0.57 | **<0.000** | 2.10 | 0.53 | **<0.000** | 2.10 | 0.50 | **<0.000** |
| $\boldsymbol{\gamma\delta}$ IRP CM Th | 0.07 | 0.07 | 1.00 | 0.12 | 0.05 | 0.33 | 0.11 | 0.02 | **0.007** |
| $\boldsymbol{\gamma\delta}$ IRP EM Th | 0.20 | 0.07 | **0.01** | 0.27 | 0.04 | **0.001** | 0.18 | 0.05 | **0.002** |
| FasL+ Th | 7.94 | 10.04 | 0.55 | 3.52 | 14.37 | **0.0001** | 2.85 | 12.49 | **0.0002** |

**Table 9.** Increased populations of cytotoxic T lymphocytes in IPF**.**

| Population | before | | | 1^st^ | | | 2^nd^ | | |
| --- | --- | --- | --- | --- | --- | --- | --- | --- | --- |
|  | Median (%) | | *P* value | Median (%) | | *P* value | Median (%) | | *P* value |
|  | IPF | control |  | IPF | control |  | IPF | control |  |
| T cytotoxic | 23.68 | 12.51 | **0.0001** | 32.79 | 12.58 | **0.0002** | 22.45 | 13.24 | **0.002** |
| Th/Tc ratio | 2.18 | 5.01 | **0.009** | 1.55 | 4.90 | **0.002** | 1.87 | 4.29 | **0.01** |
| Recent emigrants Tc | 1.23 | 0.66 | **0.02** | 0.50 | 0.50 | 0.81 | 0.86 | 0.23 | **0.02** |
| Naïve Tc | 2.87 | 2.73 | 0.47 | 4.19 | 2.54 | 0.19 | 3.63 | 2.18 | 0.20 |
| CM Tc | 2.16 | 0.49 | **<0.000** | 2.47 | 0.71 | **<0.000** | 2.80 | 0.52 | **<0.000** |
| EM Tc | 6.53 | 0.78 | **<0.000** | 7.28 | 1.07 | **<0.000** | 6.32 | 1.05 | **<0.000** |
| TEMRA Tc | 11.65 | 6.19 | **0.05** | 17.16 | 6.65 | **0.02** | 14.64 | 6.80 | 0.29 |

**Table 10.** Differentiation/activation and senescence related markers on Tc cells in IPF.

| Population | before | | | 1^st^ | | | 2^nd^ | | |
| --- | --- | --- | --- | --- | --- | --- | --- | --- | --- |
|  | Median (%) | | *P* value | Median (%) | | *P* value | Median (%) | | *P* value |
|  | IPF | control |  | IPF | control |  | IPF | control |  |
| IRP Tc | 14.03 | 3.76 | **0.003** | 16.11 | 3.93 | **0.001** | 14.36 | 4.46 | **0.005** |
| IRP CM Tc | 0.66 | 0.10 | **<0.000** | 0.71 | 0.11 | **<0.000** | 0.69 | 0.07 | **<0.000** |
| IRP EM Tc | 2.65 | 0.19 | **<0.000** | 2.36 | 0.30 | **<0.000** | 2.38 | 0.26 | **0.0001** |
| IRP TEMRA Tc | 7.30 | 2.69 | 0.06 | 10.16 | 2.97 | **0.006** | 9.78 | 3.71 | 0.10 |
| CD27+ Tc | 2.64 | 0.61 | **0.002** | 0.31 | 0.20 | 0.43 | 0.26 | 0.14 | 0.13 |
| Naïve CD27+ Tc | 0.46 | 0.16 | **0.03** | 0.02 | 0.07 | 0.35 | 0.05 | 0.04 | 0.91 |
| CM CD27+ Tc | 0.29 | 0.06 | **0.009** | 0.08 | 0.04 | 0.31 | 0.09 | 0.02 | 0.08 |
| EM CD27+ Tc | 0.39 | 0.07 | **0.003** | 0.08 | 0.06 | 0.40 | 0.07 | 0.00 | 0.07 |
| TEMRA CD27+ Tc | 0.67 | 0.21 | **0.01** | 0.16 | 0.07 | 0.25 | 0.12 | 0.05 | 0.08 |
| CD27- Tc | 16.35 | 15.39 | 0.59 | 27.69 | 13.79 | 0.06 | 24.10 | 16.73 | 0.25 |
| CD28+ Tc | 4.79 | 1.53 | **0.003** | 0.90 | 1.43 | 0.53 | 1.11 | 0.97 | 0.81 |
| CD57+ Tc | 17.93 | 11.67 | 0.28 | 26.82 | 10.27 | **0.03** | 25.06 | 11.92 | 0.09 |
| MAIT Tc | 5.45 | 4.26 | 0.50 | 2.81 | 3.33 | 0.56 | 3.18 | 2.91 | 0.59 |
| Recent emigrant MAIT Th | 0.62 | 0.26 | 0.56 | 0.11 | 0.20 | 0.13 | 0.13 | 0.14 | 0.68 |
| MAIT naïve Tc | 0.98 | 1.02 | 0.56 | 0.59 | 0.64 | 0.31 | 0.64 | 0.61 | 0.65 |
| MAIT CM Tc | 0.36 | 0.08 | **<0.000** | 0.21 | 0.14 | 0.11 | 0.69 | 0.39 | 0.19 |
| MAIT EM Tc | 0.70 | 0.23 | **<0.000** | 0.40 | 0.21 | **0.003** | 0.52 | 0.13 | **0.001** |
| MAIT TEMRA Tc | 2.46 | 2.06 | 0.56 | 1.61 | 2.41 | 0.48 | 2.07 | 2.13 | 0.81 |
| IRP MAIT Tc | 2.06 | 1.31 | 0.39 | 1.39 | 1.30 | 0.95 | 1.41 | 1.47 | 0.59 |
| IRP MAIT CM Tc | 0.06 | 0.00 | **0.0007** | 0.05 | 0.01 | 0.11 | 0.04 | 0.00 | **<0.000** |
| IRP MAIT EM Tc | 0.27 | 0.02 | **0.001** | 0.14 | 0.02 | **0.02** | 0.10 | 0.02 | **0.003** |
| IRP MAIT TEMRA Tc | 1.24 | 1.07 | 0.42 | 1.07 | 0.95 | 0.85 | 1.04 | 1.20 | 1.00 |
| FasL+ Tc | 6.94 | 9.11 | 0.28 | 4.02 | 7.99 | **0.006** | 4.21 | 12.98 | **0.02** |

**Table 11.** γδ cytotoxic T cells in IPF.

| Population | before | | | 1^st^ | | | 2^nd^ | | |
| --- | --- | --- | --- | --- | --- | --- | --- | --- | --- |
|  | Median (%) | | *P* value | Median (%) | | *P* value | Median (%) | | *P* value |
|  | IPF | control |  | IPF | control |  | IPF | control |  |
| $\boldsymbol{\gamma\delta}$ Tc | 5.78 | 0.98 | **<0.000** | 5.86 | 1.33 | **<0.000** | 6.22 | 1.22 | **<0.000** |
| Recent emigrants $\boldsymbol{\gamma\delta}$ Tc | 0.31 | 0.03 | **<0.000** | 0.13 | 0.24 | 0.62 | 0.13 | 0.02 | **0.01** |
| $\boldsymbol{\gamma\delta}$naïve Tc | 0.63 | 0.28 | **0.02** | 0.68 | 1.49 | **0.003** | 0.79 | 0.37 | **0.03** |
| $\boldsymbol{\gamma\delta}$ CM Tc | 0.44 | 0.03 | **<0.000** | 0.36 | 0.05 | **<0.000** | 0.40 | 0.04 | **<0.000** |
| $\boldsymbol{\gamma\delta}$ EM Tc | 1.38 | 0.08 | **<0.000** | 1.70 | 0.10 | **<0.000** | 1.47 | 0.06 | **<0.000** |
| $\boldsymbol{\gamma\delta}$TEMRA Tc | 2.76 | 0.48 | **<0.000** | 3.17 | 0.92 | **<0.000** | 3.33 | 0.60 | **<0.000** |
| $\boldsymbol{\gamma\delta}$ IRP CM Tc | 0.12 | 0.02 | **<0.000** | 0.12 | 0.01 | **<0.000** | 0.19 | 0.00 | **<0.000** |
| $\boldsymbol{\gamma\delta}$ IRP EM Tc | 0.86 | 0.02 | **<0.000** | 0.81 | 0.05 | **<0.000** | 0.78 | 0.02 | **<0.000** |
| $\boldsymbol{\gamma\delta}$ IRP TEMRA Tc | 2.07 | 0.26 | **<0.000** | 2.18 | 0.57 | **<0.000** | 2.38 | 0.34 | **<0.000** |

**Table 12.** Alterations in B cells phenotypes in IPF patients after vaccination course.

| Population | before | | | 1^st^ | | | 2^nd^ | | |
| --- | --- | --- | --- | --- | --- | --- | --- | --- | --- |
|  | Median (%) | | *P* value | Median (%) | | *P* value | Median (%) | | *P* value |
|  | IPF | control |  | IPF | control |  | IPF | control |  |
| CD5+ | 11.54 | 21.89 | **0.03** | 9.26 | 13.46 | 0.15 | 8.75 | 11.58 | 0.25 |
| CD5- | 89.21 | 80.38 | **0.03** | 91.17 | 87.02 | 0.19 | 91.70 | 89.83 | 0.25 |
| RBME | 3.13 | 7.32 | **0.02** | 3.87 | 6.12 | 0.18 | 2.86 | 5.29 | **0.03** |
| Memory B | 28.01 | 30.21 | 0.94 | 29.37 | 29.44 | 0.74 | 34.86 | 26.66 | **0.03** |
| Marginal zone | 16.44 | 10.80 | 0.20 | 18.69 | 10.67 | 0.08 | 15.90 | 11.45 | **0.007** |
| Switched B | 9.88 | 12.60 | 0.50 | 7.40 | 11.11 | 0.86 | 9.66 | 9.38 | 0.53 |
| Memory IgM | 0.54 | 0.92 | 0.35 | 0.81 | 0.53 | 0.26 | 0.95 | 0.88 | 0.50 |
| Naïve B | 69.88 | 65.83 | 0.85 | 69.47 | 70.56 | 0.77 | 63.39 | 72.81 | **0.05** |
| Bregs | 2.08 | 4.53 | **0.02** | 3.65 | 3.64 | 0.89 | 3.99 | 3.66 | 0.53 |
| Plasmablasts | 9.18 | 7.92 | 0.18 | 8.61 | 6.51 | 0.45 | 7.51 | 5.78 | 0.19 |

**Table 13.** NK cells

| Population | before | | | 1^st^ | | | 2^nd^ | | |
| --- | --- | --- | --- | --- | --- | --- | --- | --- | --- |
|  | Median (%) | | *P* value | Median (%) | | *P* value | Median (%) | | *P* value |
|  | IPF | control |  | IPF | control |  | IPF | control |  |
| CD3-CD56brightCD16- | 0.69 | 2.01 | **<0.000** | 0.44 | 1.62 | **<0.000** | 0.90 | 1.72 | 0.10 |
| CD3-CD56+CD16+ | 34.70 | 39.96 | 0.37 | 26.44 | 39.50 | 0.38 | 50.56 | 41.28 | 0.45 |
| CD3-CD56+CD16+CD57+ | 29.59 | 25.47 | 0.78 | 22.38 | 24.37 | 0.68 | 27.12 | 24.27 | 0.62 |
| CD3-CD56-CD16+ | 2.91 | 1.89 | 0.44 | 2.95 | 3.46 | 0.81 | 2.13 | 2.19 | 0.31 |
| CD3-CD56lowCD16- | 6.90 | 8.19 | 0.95 | 10.49 | 6.07 | 0.33 | 5.83 | 5.28 | 0.78 |

***Supplementary results***

**Differentiation of Tregs**

The overall proportions of naïve Tregs did not differ between patients and healthy individuals (1.61% vs 2.19%; p=0.91 before vaccination; 1.97% vs 2.11%; p=0.60 after the 1^st^ dose; 3.60% vs 2.34%; p=0.08 after the 2^nd^ dose), but the proportions of cells increased in the IPF group after vaccination (p=0.04) (Table 4., supplementary data).

**ICOS+PD-1+ Tregs**

We have analyzed cells that co-express inducible T cell co-stimulator (ICOS) and programmed cell death protein 1 (PD-1) receptors. After the 1^st^ (0.81% vs 0.45%; p=0.40) as well as the 2^nd^ (1.09% vs 0.74%; p=0.27) vaccination, the cell ratios in IPF patients and control subjects were comparable (Heat map 1., Table 5.; supplementary data). We did not observe alterations between IPF patients and controls when analyzing: naïve (0.05% vs 0.22%; p=0.46 before the vaccination; 0.12% vs 0.12%; p=0.74 after the 1^st^ dose; 0.24% vs 0.18%; p=0.40 after the 2^nd^ dose), central memory (0.06% vs 0.08%; p=0.91 before the vaccination; 0.08% vs 0.07%; p=0.22 after the 1^st^ dose; 0.10% vs 0.08%; p=0.17 after the 2^nd^ dose) or effector memory (0.15% vs 0.17%; p=0.50 before the vaccination; 0.31% vs 0.17%; p=0.12 after the 1^st^ dose; 0.38% vs 0.22%; p=0.07 after the 2^nd^ dose) ICOS+PD-1+ Tregs (Heat map 1., Table 5.; supplementary data).

**Follicular Tregs**

We have determined the proportions of Tregs with follicular phenotype - the ratios did not differ between IPF patients and healthy volunteers (0.02% vs 0.01%; p=0.58 before the vaccination; 0.04% vs 0.00%; p 0.20 after the 1^st^ dose; 0.06% vs 0.06%; p=0.29 after the 2^nd^ dose). However, the proportion of cells increased in IPF group after the vaccination (p=0.03). Similarly, the proportions of follicular Tregs during different stages of differentiation were comparable between IPF and healthy volunteers when analyzing: naïve follicular Tregs (0.00% vs 0.00%; p=0.71 before the vaccination; 0.00% vs 0.00%; p=0.23 after the 1^st^ dose; 0.01% vs 0.00%; p=0.17 after the 2^nd^ dose), CM follicular Tregs (0.00% vs 0.00%; p=0.71 before the vaccination; 0.00% vs 0.00%; p= 0.47 after the 1^st^ dose; 0.00% vs 0.00%; p=0.68 after the 2^nd^ dose) or EM follicular Tregs (0.01% vs 0.00%; p=0.46 before the vaccination; 0.02% vs 0.00%; p=0.07 after the 1^st^ dose; 0.04% vs 0.01%; p=0.07 after the 2^nd^ dose) However, the ratios of naïve and effector memory Tregs with follicular phenotype were heightened in IPF group after the vaccine administration (p=0.03). Proportions of effector memory follicular cells were also increased in healthy subjects (p=0.02)(Heat map 1., Table 5.; supplementary data).

**Teffs**

The ratios of effector T lymphocytes (Teffs) within the T helper population did not differ between IPF patients and healthy volunteers before vaccine administration (70.47% vs 71.06%; p=0.77). The proportions of CM effector T lymphocytes did not differ between the groups before (17.28% vs 17.59%; p=0.41) and after the 1^st^ vaccine dose (15.68% vs 12.98%; p=0.25).

**Follicular Teffs**

The analyses of follicular Teffs proven similar proportions between groups (0.04% vs 0.08%; p=0.63 before the vaccination; 0.07% vs 0.11%; p=0.33 after 2^nd^ dose). Moreover, comparable results between groups have been obtained for: naïve (0.01% vs 0.03%; p=0.18 before the vaccination; 0.03% vs 0.05%; p=0.43 after the 2nd dose), CM (0.03% vs 0.04%; p=0.91 before the vaccination; 0.05% vs 0.06%; p=0.95 after the 2nd dose) or EM (0.05% vs 0.04; p=0.26 before the vaccination; 0.12% vs 0.07%; p=0.27 after the 2nd dose) follicular Teffs (Table 7.; supplementary data).

**Differentiation of Th cells in IPF**

The proportions of helper T cells (Th) within CD3+ population were comparable when analyzing IPF patients and healthy volunteers (57.69% vs 61.37%; p=0.71 before the vaccination; 43.88% vs 60.04%; p=0.07 after the 1st dose; 51.11% vs 59.93; p=0.56 after the 2^nd^ dose).

We have also analyzed Th cells at different stages of differentiation. Before the vaccination, the proportions of Th cells that display phenotype of recent thymic emigrants ("truly naïve" cells) were increased in patients when compared to control (1.79% vs 0.60%; p=0.007). The differences were no longer significant after the vaccine administration (0.55% vs 0.84%; p=0.20 after the 1^st^ dose; 0.95% vs 0.50%; p=0.25 after the 2^nd^ dose). Proportions of these cells were increased in IPF group after the vaccination (p=0.002). On the other hand, the proportions of naïve Th cells were lower in IPF group at all time points (11.65% vs 19.66%; p=0.01 before the vaccination; 8.83% vs 19.61%; p=0.001 after the 1^st^ dose; 13.1% vs 23.95%; p=0.02 after the 2^nd^ dose). The ratios of central memory helper T lymphocytes within CD3+ cells were comparable between both groups (16.57% vs 14.83%; p=0.50 before the vaccination; 11.31% vs 10.32%; p=0.88 after the 1^st^ dose; 15.82% vs 9.81; p=0.16 after the 2^nd^ dose), whereas the proportions of effector memory cells were heightened in IPF sufferers (18.98% vs 9.01%; p=0.006 before the vaccination; 14.27% vs 9.76%; p=0.06 after the 1^st^ dose; 14.03% vs 9.72%; p=0.02 after the 2^nd^ dose)(Heat map 2., Table 8.; supplementary data).

**Activation and senescence related markers on Th cells in IPF**

We have also analyzed several markers connected with T cells' activation and senescence. Firstly, we have evaluated presence of CD27 receptor that serves as co-stimulatory immune checkpoint molecule. The proportions CD27-positive (17.82% vs 9.12%; p=0.34 before the vaccination; 0.64% vs 2.51%; p=0.29 after the 1^st^ dose; 0.58% vs 1.37%; p=0.85 after the 2^nd^ dose) as well as CD27-negative (10.38% vs 14.25%; p=0.24 before the vaccination; 16.04% vs 24.16%; p=0.08 after the 1^st^ dose; 14.24% vs 18.19%; p=0.16 after the 2^nd^ dose) Th cells were comparable between groups. The ratios of CD27+ Th cells were reduced in patients after the vaccination (p=0.0003). Moreover, we have not observed differences in the ratios of: naïve (6.56% vs 4.65%; p=0.86 before the vaccination; 0.41% vs 1.94%; p=0.14 after the 1^st^ dose; 0.69% vs 0.81%; p=0.86 after the 2^nd^ dose), central memory (7.71% vs 3.77%; p=0.35 before the vaccination; 0.30% vs 2.39%; p=0.19 after the 1^st^ dose; 0.77% vs 1.27%; p=0.78 after the 2^nd^ dose), as well as effector memory (2.72% vs 1.94%; p=0.31 before the vaccination; 0.39% vs 1.06%; p=0.45 after the 1^st^ dose; 0.51% vs 0.63%; p=0.91 after the 2^nd^ dose) CD27+ Th cells. Simultaneously, the proportions of naïve and central memory CD27+ Th cells decreased in IPF sufferers after the vaccination (p<0.000 for all)(Table 8., supplementary data).

Secondly, we have evaluated the proportions of Th cells that exhibited the expression of CD28 molecule – co-stimulatory receptor required for T cells activation. The ratios of CD28+Th/CD3+ were similar between IPF and healthy subjects before the vaccination (7.42% vs 3.93%; p=0.37) but decreased in patients afterwards (0.50% vs 3.08%; p=0.0006 after the 1^st^ dose; 0.73% vs 1.73%; p=0.04 after the 2^nd^ dose). Vaccination was connected with decreased proportions of these cells in IPF (p=0.001) as well as control group (p=0.045).

We have also assessed the presence of CD57 molecule on T cells as a marker of cellular senescence and absence of proliferative capacity. The overall proportions of CD57+ Th cells were comparable between groups (3.65% vs 3.39%; p=1.00 before the vaccination; 4.23% vs 3.84%; p=0.98 after the 1^st^ dose; 3.85% vs 3.37%; p=0.81 after the 2^nd^ dose).

When analysing overall proportions of CD28-CD57+ senescent Th cells within CD3+ population, we have obtained similar results for IPF and healthy group (2.79% vs 3.23%; p=0.44 before the vaccination; 5.01% vs 2.92%; p=0.43 after the 1^st^ dose; 2.91% vs 3.06%; p=0.88 after the 2^nd^ dose). However, the proportions significantly increased in IPF group after the vaccination (p=0.013). The proportions of CM CD28-CD57+ Th lymphocytes were similar between groups (0.42% vs 0.46%; p=0.88 before the vaccination; 0.74% vs 0.47%; p=0.40 after the 1^st^ dose; 0.65% vs 0.35%; p=0.06 after the 2^nd^ dose). Similar results have been observed for EM CD28-CD57+ helper T cells (1.33% vs 0.59%; p=0.11 before the vaccination; 2.21 % vs 0.54%; p=0.01 after the 1^st^ dose; 1.36% vs 0.63%; p=0.17 after the 2^nd^ dose). IPF group displayed heightened proportions of CD28-CD57+ cells with central memory phenotype after the vaccination (p=0.045).

We have also determined proportions of FasL+ Th cells - population with possible cytotoxic activity. The prevalence of these lymphocytes was similar between IPF and healthy subjects before the vaccination (7.94% vs 10.04%; p=0.55), but comparison performed after vaccine administration revealed decreased proportions in patients (3.52% vs 14.37; p=0.0001 after the 1^st^ dose; 2.85% vs 12.49%; p=0.0002 after the 2^nd^ dose). Ratios of FasL+CD3+CD4+/CD3+ decreased in IPF sufferers after the vaccine administration (p=0.02)(Heat map 3., Table 8.; supplementary data).

**Impairment of MAIT CD4+ cells in IPF**

Our group has also performed analysis of mucosal-associated invariant T (MAIT) CD4+ lymphocytes, characterized by the presence of TCR Vα7.2 chain. The overall proportions of MAIT helper T cells between groups were similar (2.61% vs 4.61%; p=0.06 before the vaccination; 2.52% vs 3.21%; p=0.53 after the 2^nd^ dose) (Figure 2.A., Table 8.; supplementary data). However, the proportions of MAIT Th/CD3+ significantly increased in IPF group after the vaccination (p=0.049).

The proportions of recent thymic emigrants of MAIT Th cells did not differ between groups (0.23% vs 0.17%; p=0.59 before the vaccination; 0.06% vs 0.17%; p=0.02 after the 1^st^ dose; 0.07% vs 0.10%; p=0.43 after the 2^nd^ dose), however, patients displayed reduced ratios of these cells after the vaccination (p=0.001). The ratios of naïve MAIT CD4+ cells within CD3+ population were lowered in IPF patients when compared to healthy subjects (1.09% vs 1.62%; p=0.04 before the vaccination; 0.05% vs 1.54%; p=0.001 after the 1^st^ dose; 0.83% vs 1.35%; p=0.07 after the 2^nd^ dose) and decreased in patients after the vaccination (p=0.004). The proportions of CM (0.48% vs 0.80%; p=0.24 before the vaccination; 0.63% vs 0.67%; p=0.40 after the 1^st^ dose; 0.68% vs 0.35%; p=0.11 after the 2^nd^ dose) as well as EM (0.59% vs 0.38%; p=0.18 before the vaccination; 0.59% vs 0.48%; p=0.33 after the 1^st^ dose; 0.61% vs 0.22%; p=0.06 after the 2^nd^ dose) MAIT Th cells were similar between groups.

We have also explored the presence of CD28-CD57+ senescent phenotype on MAIT helper T cells. The overall proportions of cells were reduced in IPF patients before the vaccination (0.16% vs 0.33%; p=0.04), but not after (0.02% vs 0.07%; p=0.23 after the 1^st^ dose; 0.30% vs 0.23%; p=0.71 after the 2^nd^ dose). Groups displayed similar proportions of senescent central memory (0.01% vs 0.02%; p=0.30 before the vaccination; 0.02% vs 0.03%; p=1.00 after the 1^st^ dose; 0.03% vs 0.01%; p=0.25 after the 2^nd^ dose) MAIT Th cells as well as their effector memory counterparts (0.05% vs 0.03%; p=0.78 before the vaccination; 0.17% vs 0.04%; p=0.12 after the 1^st^ dose; 0.10% vs 0.01%; p=0.15 after the 2^nd^ dose)(Heat map 3., Table 8.; supplementary data).

**γδ helper T Cells in IPF**

IPF patients displayed increased proportions of γδ Th (6.84% vs 4.04%; p=0.02 before the vaccination; 6.74% vs 1.22%; p=0.007 after the 2^nd^ dose)(Figure 2.B., Table 8.; supplementary data).

The populations of truly naïve γδ Th cells were heightened in IPF before the vaccination (0.24% vs 0.10%; p=0.03). However, the ratios were similar after vaccine administration (0.09% vs 0.15%; p=0.40 after the 1^st^ dose; 0.16% vs 0.09%; p=0.23 after the 2^nd^ dose). The ratios of naïve γδ Th lymphocytes within CD3+ population did not differ between groups (1.54% vs 1.31%; p=0.80 before the vaccination; 0.98% vs 1.90%; p=0.22 after the 1^st^ dose; 1.51% vs 1.68%; p=1.00 after the 2^nd^ dose). The proportions of naïve cells in IPF declined after the vaccination (p=0.046). The prevalence of CM (1.36% vs 0.77%; p=0.008 before the vaccination; 1.27% vs 0.61%; p=0.05 after 1^st^ dose; 1.67% vs 0.50%; p<0.000 after 2^nd^ dose) as well as EM (2.14% vs 0.57%; p<0.000 before the vaccination; 10% vs 0.53%; p<0.000 after 1^st^ dose; 2.10% vs 0.50%; p<0.000 after 2^nd^ dose) γδ Th cells was elevated in IPF.

The proportions of senescent central memory γδ Th were comparable between IPF and healthy volunteers during initial time points (0.07% vs 0.07%; p=1.00 before vaccination; 0.12% vs 0.05%; p=0.33 after the 1^st^ dose). After vaccination the population was elevated in IPF patients (0.11% vs 0.02%; p=0.007). Ratios of senescent effector memory γδ Th lymphocytes were proven increased in IPF sufferers (0.20% vs 0.07%; p=0.01 before the vaccination; 0.27% vs 0.04%; p=0.001 after the 1^st^ dose; 0.18% vs 0.05%; p=0.002 after the 2^nd^ dose)(Heat map 3., Table 8.; supplementary data).

**T cytotoxic cells**

The ratios of naïve Tc/CD3+ were similar between the groups (2.87% vs 2.73%; p=0.47 before vaccination; 4.19% vs 2.54%; p=0.19 after the 1^st^ dose; 3.63% vs 2.18%; p=0.20 after the 2^nd^ dose).

Analysis of terminally-differentiated CD45 RA-positive effector memory Tc cells revealed lack of significant differences between the groups (11.65% vs 6.19%; p=0.05 before vaccination; 14.64% vs 6.80%; p=0.29 after the 2^nd^ dose) (Heat map 4., Table 9.; supplementary data).

**CD27-, CD28+ and CD57+ cytotoxic T Cells in IPF**

Proportions of CD27-negative cytotoxic T cells were comparable between IPF and control group (16.35% vs 15.39%; p=0.59 before the vaccination; 27.69% vs 13.79%; p=0.06 after the 1^st^ dose; 24.10% vs 16.73%; p=0.25 after the 2^nd^ dose).

CD28-positive cytotoxic T cells were heightened in IPF only before the vaccination (4.79% vs 1.53%; p=0.003). The differences were lost after the procedure (0.90% vs 1.43%; p=0.53 after the 1^st^ dose; 1.11% vs 0.97%; p=0.81 after the 2^nd^ dose) as proportions of cells were significantly reduced in patients after the vaccination (p=0.005).

Inquiries concerning CD57+ cytotoxic T cells indicated lack of significant differences between IPF and control group (17.93% vs 11.67%; p=0.28 before the vaccination; 25.06% vs 11.92%; p=0.09 after 2^nd^ dose). Analysis of variance suggested increase of cells in IPF patients after the vaccination (p=0.006)(Heat map 4., Table 9.; supplementary data).

The proportions of CD28-CD57+ TEMRA cells did not differ between the groups (7.30% vs 2.69%; p=0.06 before vaccination; 9.78% vs 3.71%; p=0.10 after the 2^nd^ dose).

**MAIT CD8+ cells in IPF**

Evaluation of overall population of TCR Vα7.2+ mucosal-associated invariant T (MAIT) CD8+ lymphocytes have shown lack of differences between IPF and control subjects (5.45% vs 4.26%; p=0.50 before the vaccination; 2.81% vs 3.33%; p=0.56 after the 1^st^ dose; 3.18% vs 2.91%; p=0.59 after the 2^nd^ dose) (Figure 2.C., Table 10; supplementary data).

Similarly, the ratios of recent thymic emigrants (0.62% vs 0.26%; p=0.56 before the vaccination; 0.11% vs 0.20%; p=0.13 after the 1^st^ dose; 0.13% vs 0.14%; p=0.68 after the 2^nd^ dose) and naïve (0.98% vs 1.02%; p=0.56 before the vaccination; 0.59% vs 0.64%; p=0.31 after the 1^st^ dose; 0.64% vs 0.61%; p=0.65 after the 2^nd^ dose) MAIT cytotoxic T cells did not vary between groups. Ratios of true naïve MAIT Tc cells decreased in IPF group after the vaccination (p=0.002). The prevalence of central memory (0.36% vs 0.08; p<0.000 before the vaccination; 0.21% vs 0.14%; p=0.11 after the 1^st^ dose; 0.69% vs 0.39%; p=0.19 after the 2^nd^ dose) and effector memory (0.70% vs 0.23%; p<0.000 before the vaccination; 0.40% vs 0.21%; p=0.003 after the 1^st^ dose; 0.52% vs 0.13%; p=0.001 after the 2^nd^ dose) MAIT cells were increased in IPF. The proportions of terminally-differentiated MAIT Tc lymphocytes were similar between patients and control (1.61% vs 2.41%; p=0.48 after the 1^st^ dose; 2.07% vs 2.13%; p=0.81 after the 2^nd^ dose).

Overall ratios of senescent CD28-CD57+ MAIT Tc lymphocytes within CD3+ cells did not differ between groups (2.06% vs 1.31.%; p=0.39 before the vaccination; 1.39% vs 1.30%; p=0.95 after the 1^st^ dose; 1.41% vs 1.47%; p=0.59 after the 2^nd^ dose). The ratios of central memory (0.06% vs 0.00%; p=0.0007 before the vaccination; 0.05% vs 0.01%; p=0.11 after the 1^st^ dose; 0.04% vs 0.00%; p<0.000 after the 2^nd^ dose) as well as effector memory (0.27% vs 0.02%; p=0.001 before the vaccination; 0.14% vs 0.02%; p=0.02 after the 1^st^ dose; 0.10% vs 0.02%; p=0.003 after the 2^nd^ dose) senescent MAIT Tc lymphocytes were heightened in IPF sufferers. The proportions of CD28-CD57+ TEMRA MAIT Tc cells were similar between both groups (1.07% vs 0.95%; p=0.85 after the 1^st^ dose; 1.04% vs 1.20%; p=1.00 after the 2^nd^ dose)(Heat map 4., Table 10.; supplementary data).

**Alterations in B cells phenotypes in IPF patients after vaccination course.**

Before the vaccination procedure, we have observed decreased percentages of CD5-positive B cells in IPF patients compared to healthy controls (11.54% vs. 21.89%; p=0.03). However, after the vaccination, the proportions of CD5+ B cells were comparable between groups (9.26% vs. 13.46%; p=0.15 after the 1^st^ dose and 8.75% vs. 11.58%; p=0.25 after the 2^nd^ dose). Similar results have been detected when analyzing CD5-negative population - before the vaccination IPF patients exhibited higher ratios of cells when contrasted with healthy volunteers (89.21% vs. 80.38%; p=0.03). After vaccine administration the percentages of CD5- cells did not differ (91.17% vs. 87.02%; p=0.19 after the 1^st^ dose; 91.70% vs. 89.93%; p=0.25 after the 2^nd^ dose)(Figure 2.D., Table 12.; supplementary data).

We have also examined the expression of classical differentiation markers on B cells. The overall proportions of naïve B lymphocytes did not differ between patients and healthy individuals before and after the 1^st^ dose of the vaccine (69.88% vs. 65.83%; p=0.85 before the vaccination; 69.47% vs. 70.56%; p=0.77 after the 1^st^ dose). However, after the vaccine procedure completion IPF patients were characterized by lower proportions of naïve B cells (63.30% vs. 72.81%; p=0.05). The percentages of memory B cells also did not differ between groups before vaccination (28.01% vs. 30.21%; p=0.94) and after the 1^st^ dose (29.37% vs. 29.44%; p=0.74). However, cells were found increased in IPF sufferers after the 2^nd^ vaccine dose (34.86% vs. 26.66%; p=0.03)(Figure 2.E., Table 12.; supplementary data). We have observed similar results during marginal zone B cells analyses – populations were similar between groups before (16.44% vs. 10.80%; p=0.20) and after 1^st^ vaccine dose (18.69% vs. 10.67%; p=0.08). Ratios of marginal zone B cells within B lymphocytes were heightened in IPF patients after vaccine procedure completion (15.90% vs. 11.45%; p=0.007). IPF sufferers displayed comparable ratios of IgM+ memory B cells (0.54% vs. 0.92%; p=0.35 before the vaccination; 0.81% vs. 0.53%; p=0.26 after the 1^st^ dose; 0.95% vs. 0.88%; p=0.50 after the 2^nd^ dose) as well as switched B cells populations (9.88% vs. 12.60%; p=0.50 before the vaccination; 7.40% vs. 11.11%; p=0.86 after the 1^st^ dose; 9.66% vs. 9.38%; p=0.53 after the 2^nd^ dose) at all time points when contrasted with healthy volunteers.

Before the vaccination, the proportions of Bregs were significantly lower in IPF patients when compared to healthy volunteers (2.08% vs. 4.53%; p=0.02). However, similar results were not observed after vaccine administration (3.65% vs. 3.64%; p=0.89 after the 1^st^ dose; 3.99% vs. 3.66%; p=0.53 after the 2^nd^ dose)(Figure 2.F., Table 12.; supplementary data).

The proportions of recent bone marrow emigrants (RBME) were decreased in IPF sufferers (3.13% vs. 7.32%; p=0.02 before the vaccination; 2.86% vs. 5.29%; p=0.03 after 2^nd^ dose). The populations of plasmablasts were comparable between both groups (9.18% vs. 7.92%; p=0.18 before the vaccination; 8.61% vs. 6.51%; p=0.45 after the 1^st^ dose; 7.51% vs. 5.78%; p=0.19 after the 2^nd^ dose)(Figure 2.G., Table 12; supplementary data).

The ratios of CD21^low^B cells did not differ between IPF patients and healthy individuals (4.17% vs. 6.78%; p=0.26 before the vaccination; 8.00% vs. 5.29%; p=0.08 after 2^nd^ dose). CD21^low^ cells significantly increased in IPF group after the vaccination (p=0.02)(Heat map 5., Table 12.; supplementary data).

**NK cells in IPF**

The proportions of CD56+CD16+ NK cells within CD3- population were comparable between IPF and healthy volunteers (34.70% vs. 39.96%; p=0.37 before the vaccination; 26.44% vs. 39.50%; p=0.38 after the 1^st^ dose; 50.56% vs. 41.28%; p=0.45 after the 2^nd^ dose)(Figure 2.H., Table 13.; supplementary data)

On the other hand, the ratios of CD56^bright^CD16- NK cells were significantly decreased in IPF patients before (0.69% vs. 2.01%; p<0.000) and after the 1^st^ (0.44% vs. 1.62%; p<0.000) vaccine dose. The differences after the vaccine completion did not reach statistical significance (0.90% vs. 1.72%; p=0.10). The proportions of CD56^bright^CD16- NK cells increased in the IPF group after the vaccination (p=0.04)(Figure 2.I., Table 13.; supplementary data).

The proportions of precursor CD56-CD16+NK lymphocytes (2.91% vs. 1.89%; p=0.44 before the vaccination; 2.95% vs. 3.46%; p=0.81 after the 1^st^ dose, and 2.13% vs. 2.19%; p=0.31 after the 2^nd^ dose) (Figure 2.J.; supplementary data) as well as mature CD57+NK cell subsets (29.59% vs. 25.47%; p=0.78 before the vaccination; 22.38% vs. 24.37%; p=0.68 after the 1^st^ dose; 27.12% vs. 24.27%; p=0.62 after the 2^nd^ dose) were comparable between IPF patients and healthy volunteers. The results obtained for CD56^low^CD16- cells were also similar when analyzing IPF and control group (6.90% vs. 8.19%; p=0.95 before the vaccination; 10.49% vs. 6.07%; p=0.33 after the 1^st^ dose; 5.83% vs. 5.28%; p=0.78 after the 2^nd^ dose)(Figure 2.K-L.; Heat map 6., Table 13.; supplementary data).

***
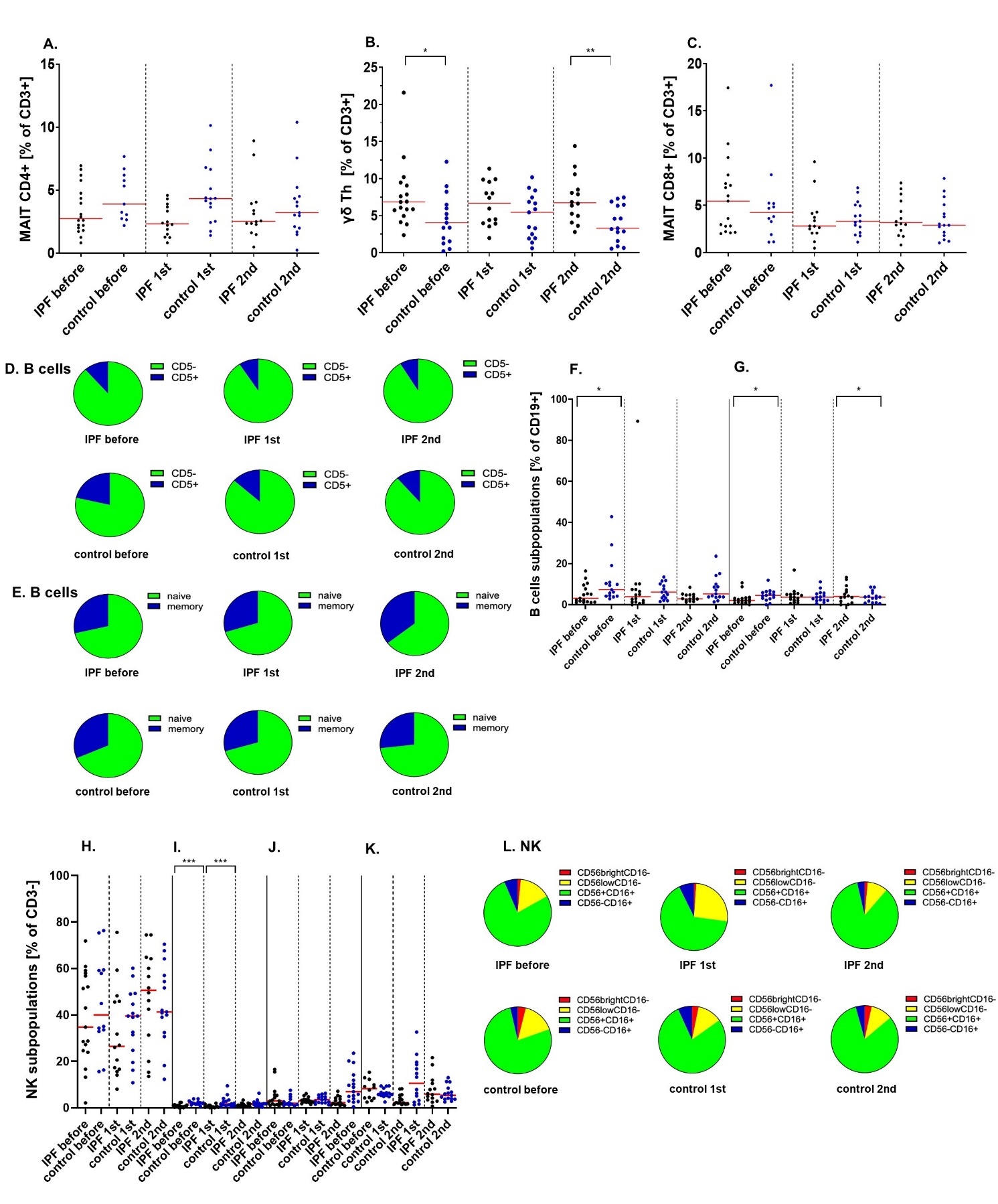
***

**Figure 2.** Illustration of the dynamic alterations in immune cell populations during the vaccine course (before, after the 1^st^ and after the 2^nd^ dose of vaccine) between two analyzed groups (IPF vs. healthy control). **(A)** Percentages of mucosal-associated invariant T (MAIT) CD4+ cells within analyzed populations during vaccination course. **(B)** Percentages of γδ helper T cells within analyzed populations during vaccination course. **(C)** Percentages of mucosal-associated invariant T (MAIT) CD8+ cells within analyzed populations during vaccination course. **(D)** Proportions of CD5- and CD5+ cells within the B lymphocyte population in IPF and control group. **(E)** Proportions of naïve and memory cells within B lymphocyte population in both groups. Percentages of Bregs **(F)** and RBME **(G)** cells within analyzed populations during vaccination course. Percentages of CD56+CD16+ **(H),** CD56^bright^CD16- **(I),** CD56-CD16+ **(J)** and CD56^low^CD16- **(K)** NK cells within analyzed populations during vaccination course. **(L)** Pie charts showing the distribution of the populations in the analyzed groups at the 3 time points: before vaccination, after 1^st^ dose and after 2^nd^ dose. Significant results are marked with * (p<0.05), ** (p<0.01), or *** (p<0.001).

**Heat map 1.** Subpopulations of T regulatory cells. Both rows and columns are clustered using Euclidean distance.


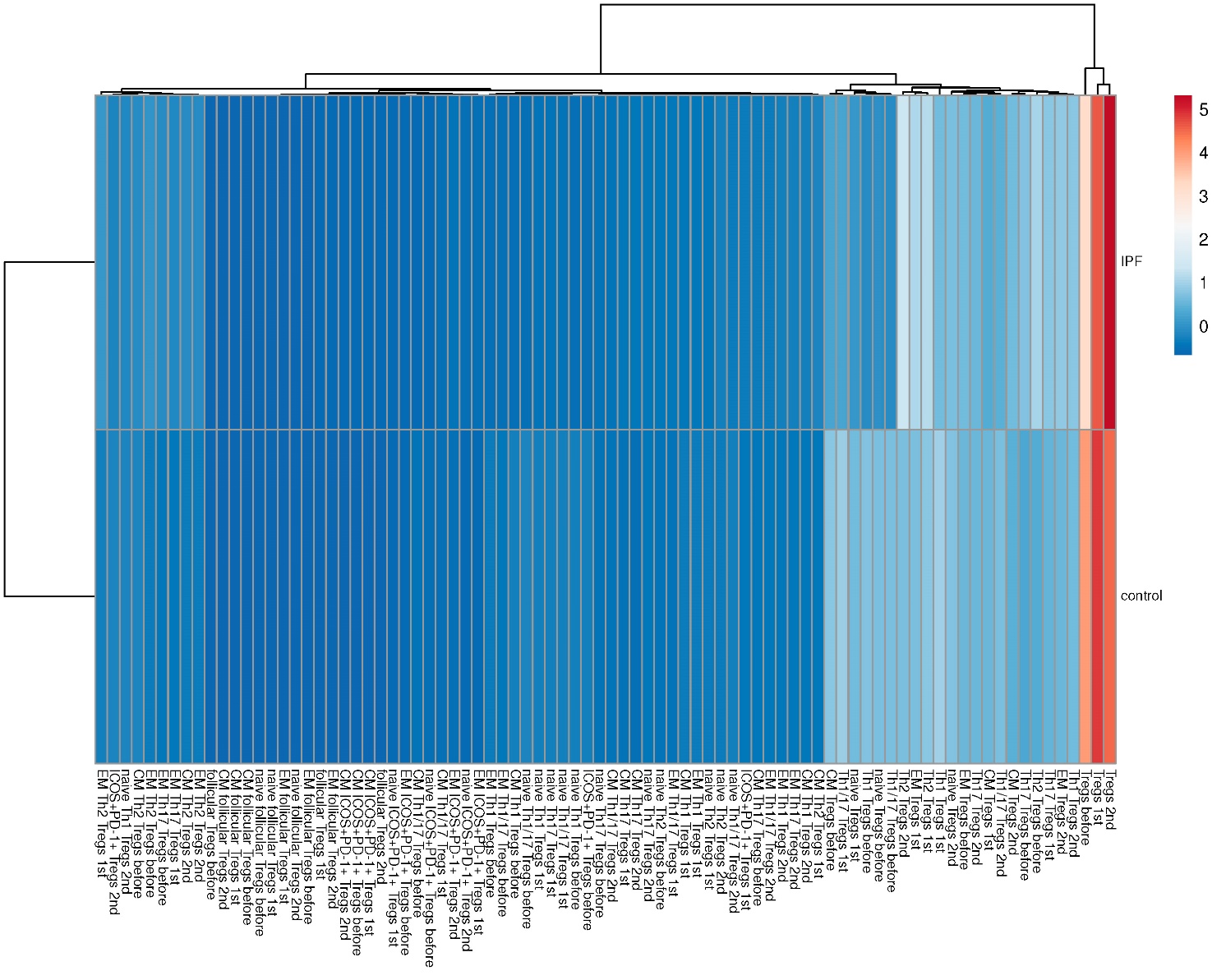


**Heat map 2.** Subpopulations of T effector cells. Both rows and columns are clustered using Euclidean distance.

**
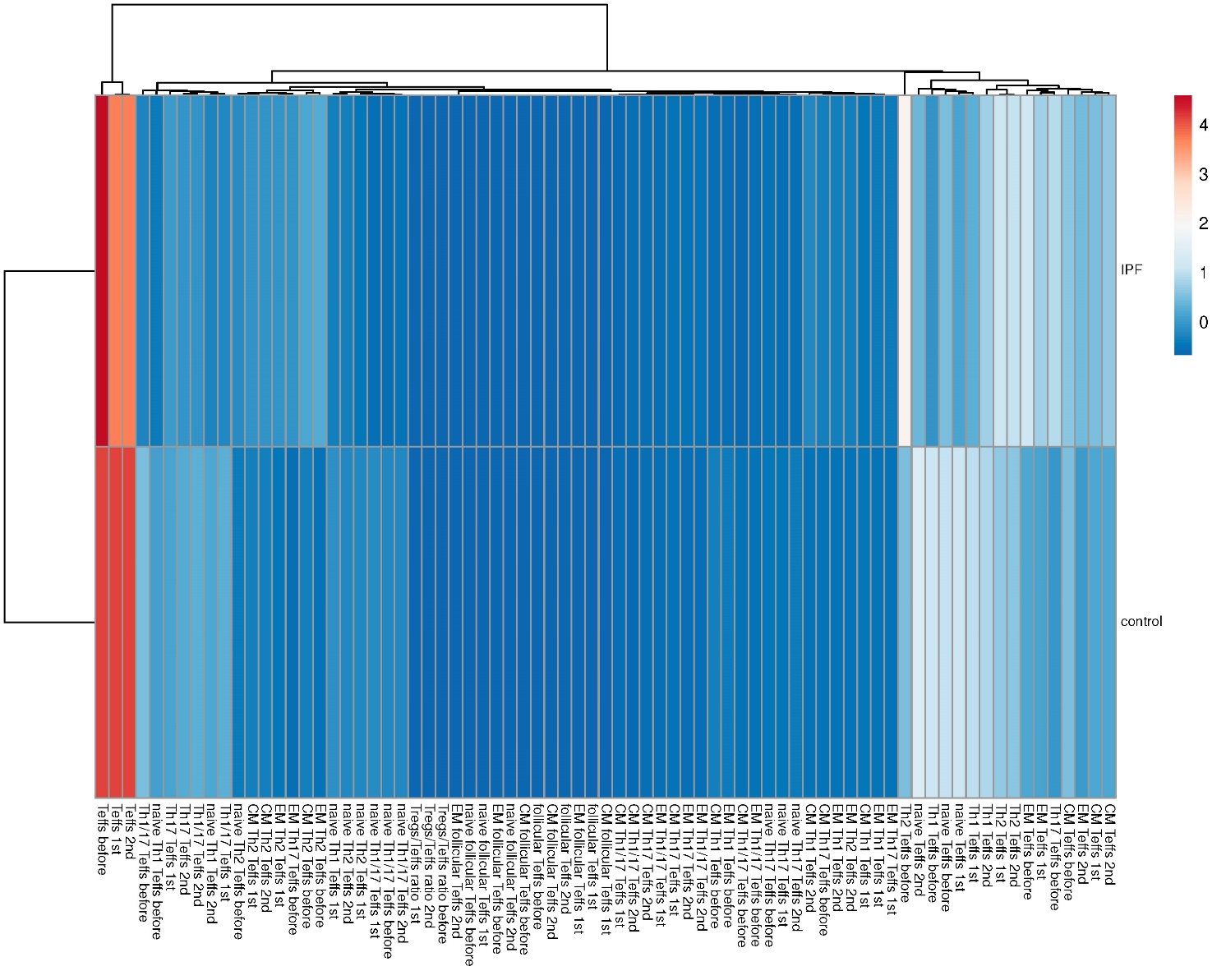
**

**Heat map 3**. Subpopulations of T helper cells. Both rows and columns are clustered using Euclidean distance.


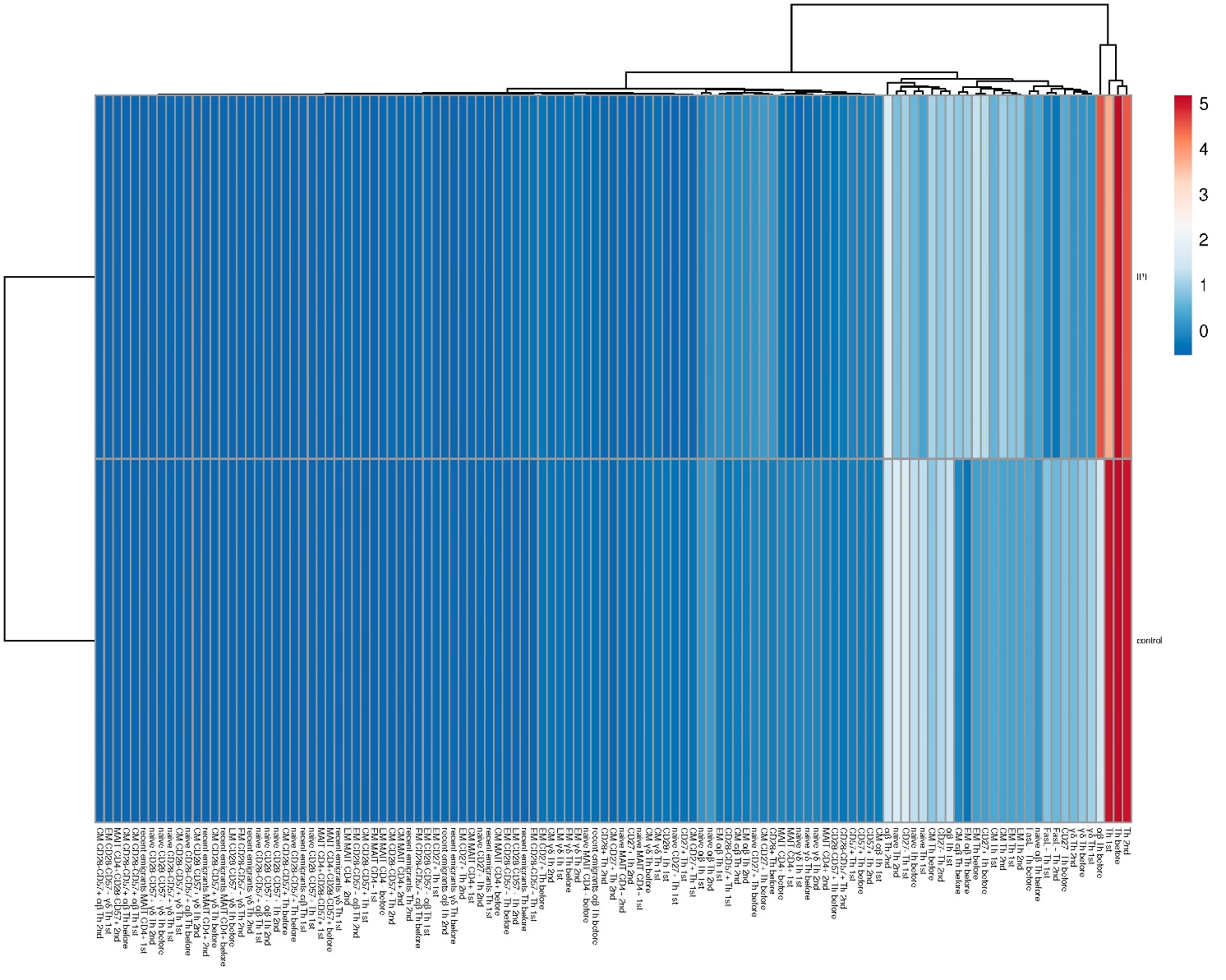


**Heat map 4.** Subpopulations of T cytotoxic cells. Both rows and columns are clustered using Euclidean distance.


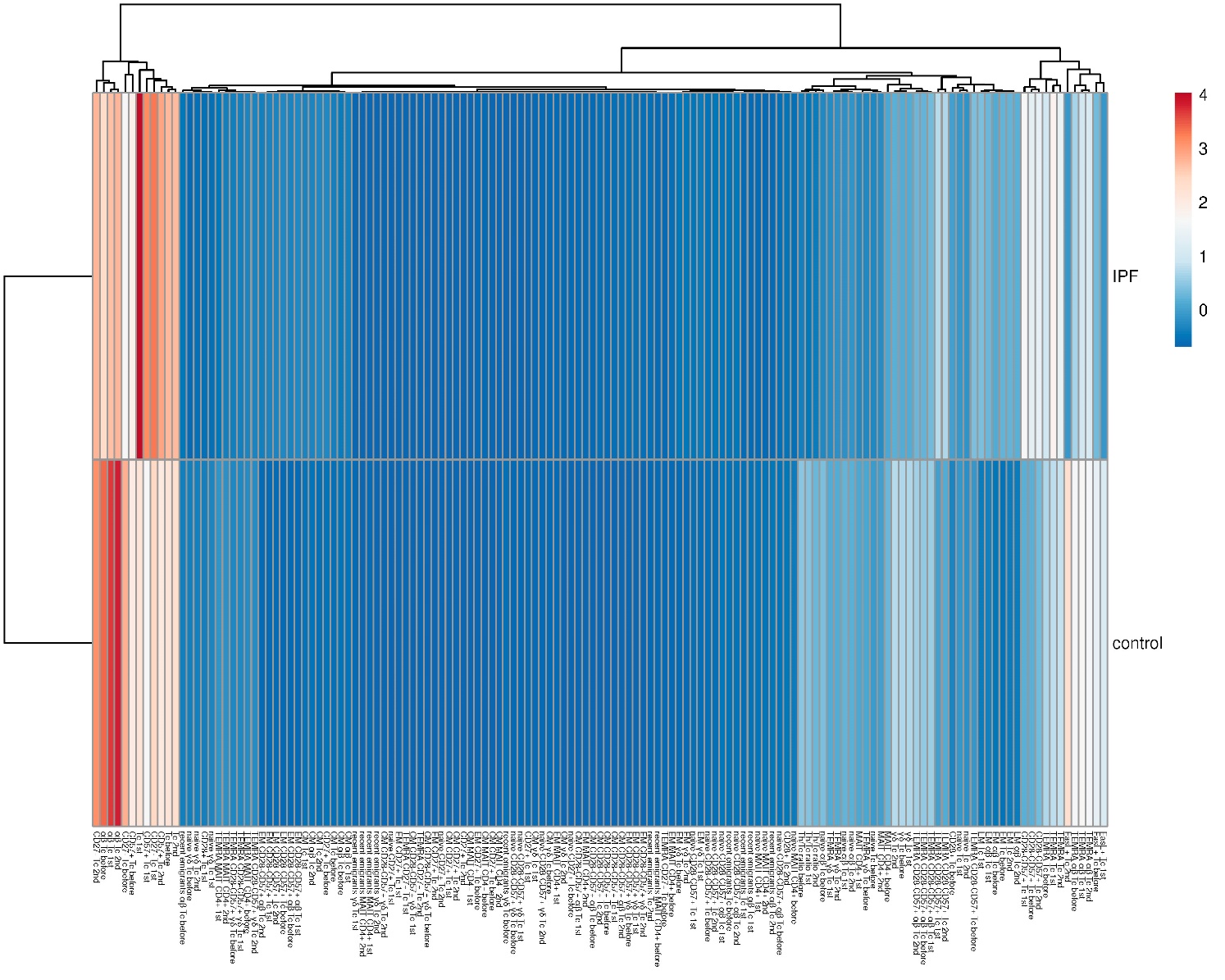


**Heat map 5.** Subpopulations of B lymphocytes. Both rows and columns are clustered using Euclidean distance.


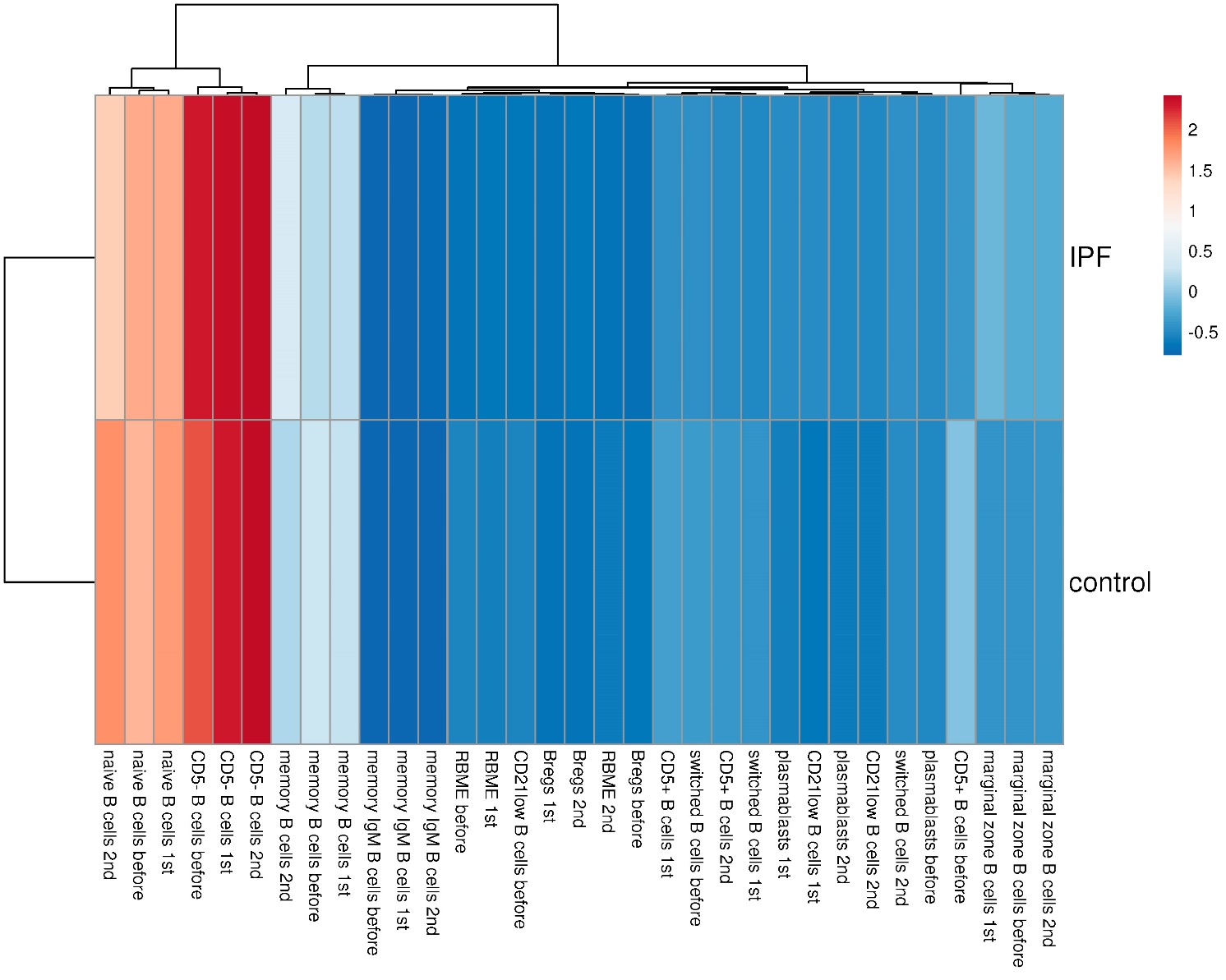


**Heat map 6.** Subpopulations of NK cells. Both rows and columns are clustered using Euclidean distance.


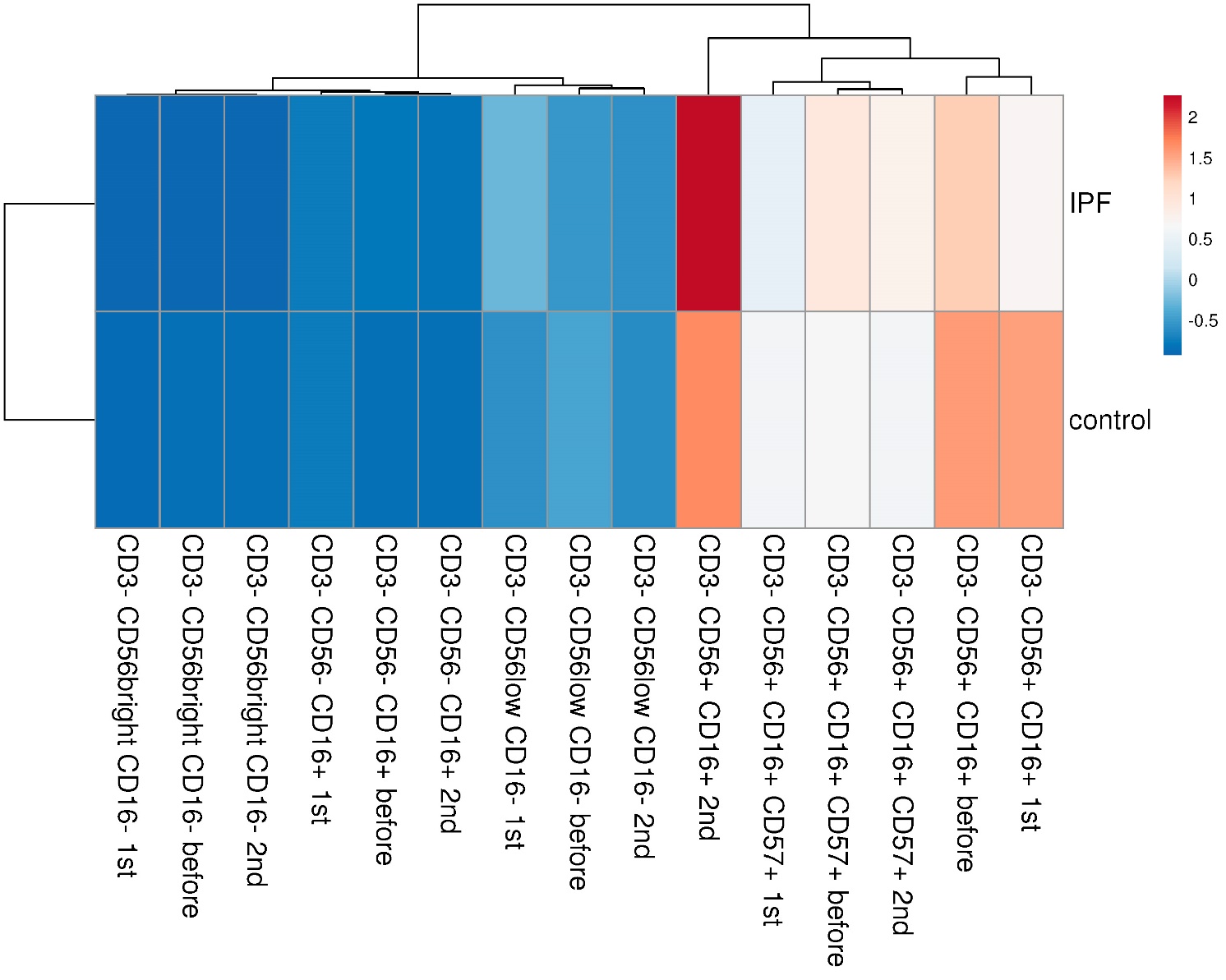


**Principal Component Analysis**

**Tregs**

Before the vaccination and after the 1^st^ vaccine dose the most discriminative subpopulations between IPF and control group within Tregs were T regulatory cells, naïve Tregs, CM Tregs as well as their Th1, Th17, Th1/17 and Th2 counterparts, based on eigenvalues ≥1 (PCA1, PCA2). After the second dose, eigenvalues ≥1 were obtained for Tregs, CM Tregs as well as Th17, Th1/17 and Th2 CM Tregs.


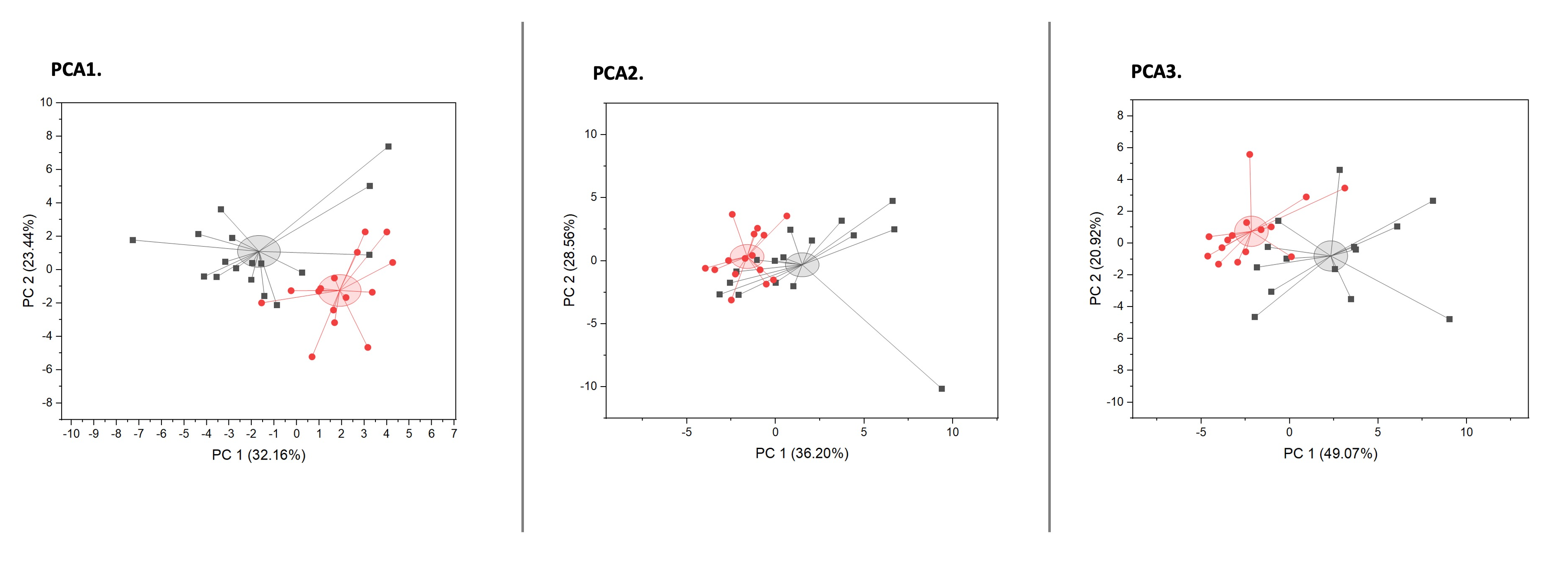


**PCA 1.** regulatory T cells in both groups before vaccination.; **PCA 2**. regulatory T cells in both groups after the 1^st^ dose of vaccine.; **PCA 3**. regulatory T cells in both groups after the 2^nd^ dose of vaccine. Each dot represents a single sample colored by group (gray - IPF, red – control).

**Teffs**

Among the effector T cells, the most distinguishing populations (eigenvalues ≥1) between studied groups in all time points were the subpopulations of central memory Th2, Th1/17 and Th17 cells. The highest eigenvalues were achieved for central memory Teffs, Teffs and Tregs/Teffs ratio.


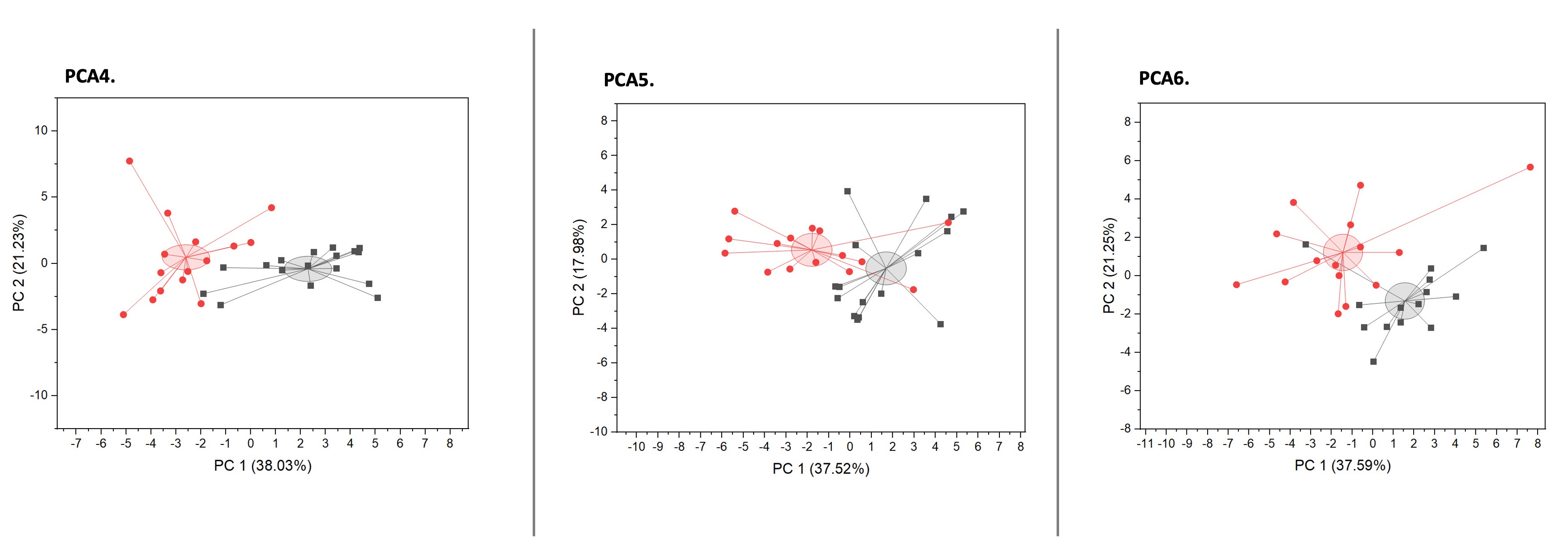


**PCA 4.** eEffector T lymphocytes in both groups before vaccination.; **PCA 5**. effector T cells in both groups after the 1^st^ dose of vaccine.; **PCA 6**. effector T cells in both groups after the 2^nd^ dose of vaccine. Each dot represents a single sample colored by group (gray - IPF, red – control).

**T helpers**

PCA analysis proved that, before the vaccine administration, the differentiating population with eigenvalues ≥1 comprised of: Th cells, naïve, CD27+ naïve, recent thymic emigrants, CM as well as CD27+ CM Th cells. Values ≥1 were obtained also for CM lymphocytes with senescent phenotype. After the 1^st^ vaccine dose, in addition to previously mentioned cells, the distinguishing population also included EM Th lymphocytes. During the third time point, the number of discriminating leukocytes included: T helpers cells, their naïve and central memory subpopulations as well as CD27+ CM Th. Also, recent thymic emigrants and senescent CM T helpers gained eigenvalues ≥1.

**
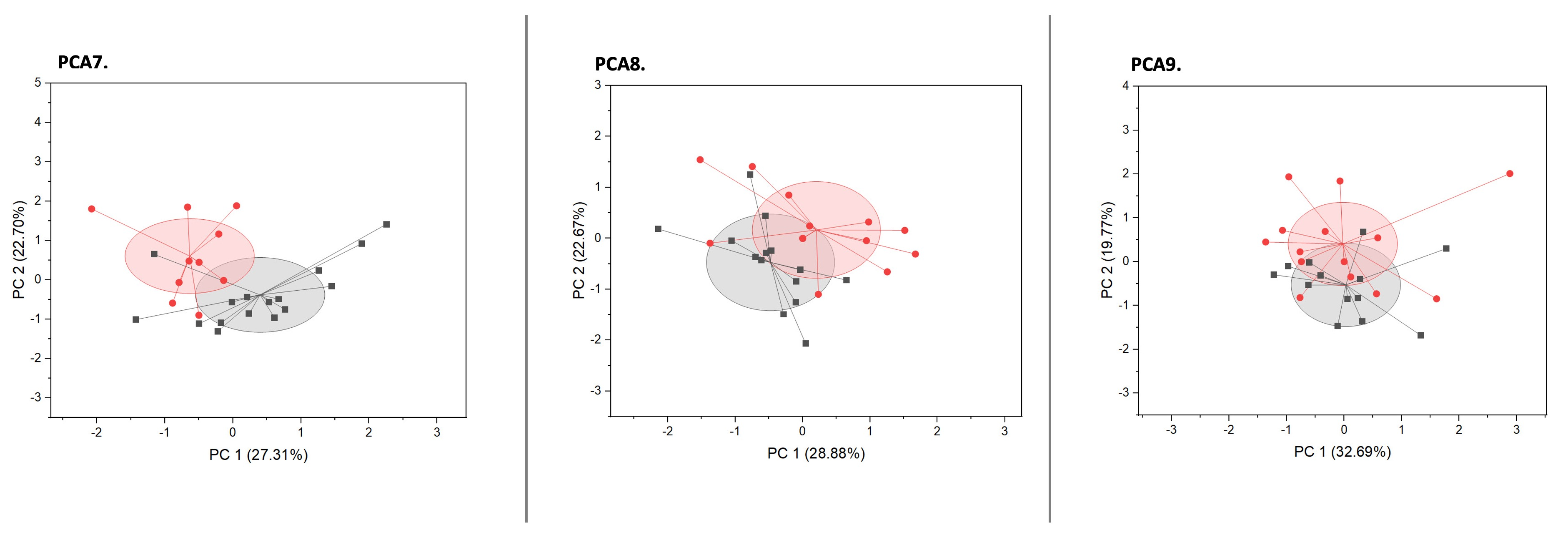
PCA 7.** helper T cells in both groups before vaccination.; **PCA 8**. helper T cells in both groups after the 1^st^ dose of vaccine.; **PCA 9**. helper T cells in both groups after the 2^nd^ dose of vaccine. Each dot represents a single sample colored by group (gray - IPF, red – control).

**T cytotoxic cells**

Within cytotoxic T cells, the discriminating populations (eigenvalues ≥1) before the vaccination included: cytotoxic T cells, naïve Tc, recent thymic emigrant Tc, CM Tc, CD27+ and senescent CM CD8+ cells. After the 1^st^ and the 2^nd^ doses, beside the above mentioned, the distinguishing lymphocytes also consisted of CD27+ naïve cells.


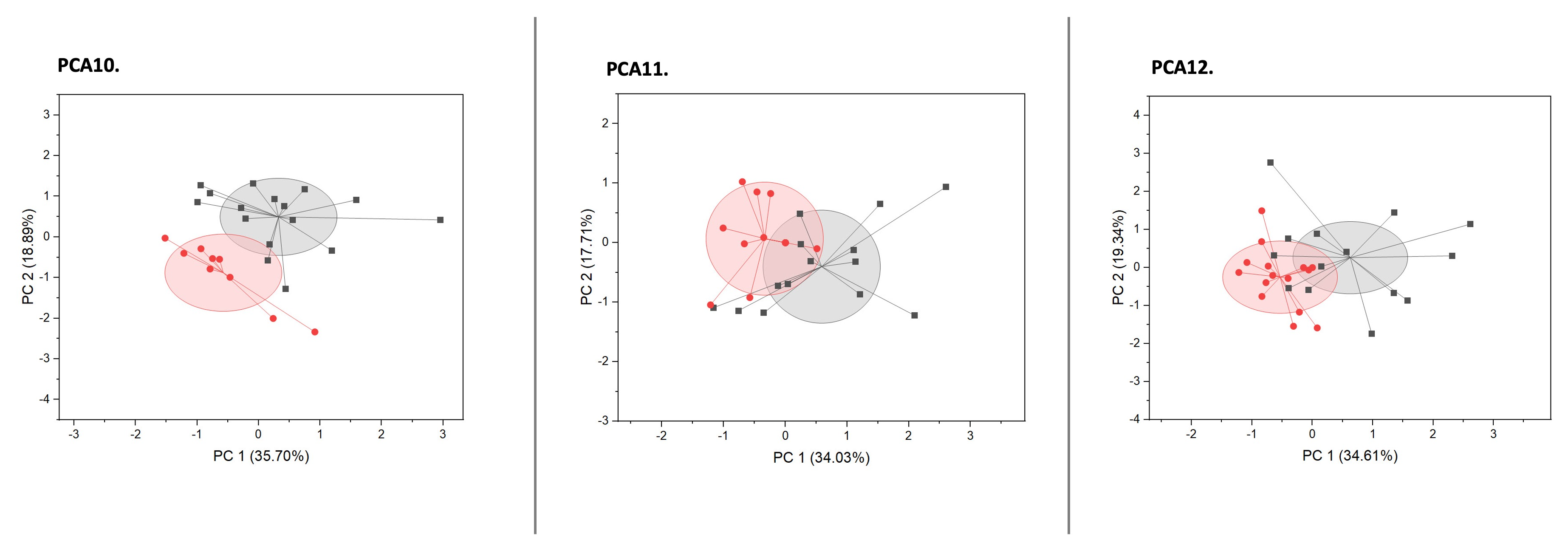


**PCA 10.** cytotoxic T cells in both groups before vaccination.; **PCA 11**. cytotoxic T cells in both groups after the 1^st^ dose of vaccine.; **PCA 12**. cytotoxic T cells in both groups after the 2^nd^ dose of vaccine. Each dot represents a single sample colored by group (gray - IPF, red – control).

**B cells**

During all analyzed time points, IPF and control group were differentiated (eigenvalues ≥1). by CD5+ as well as CD5- B lymphocytes, memory B cells and recent bone marrow emigrants.


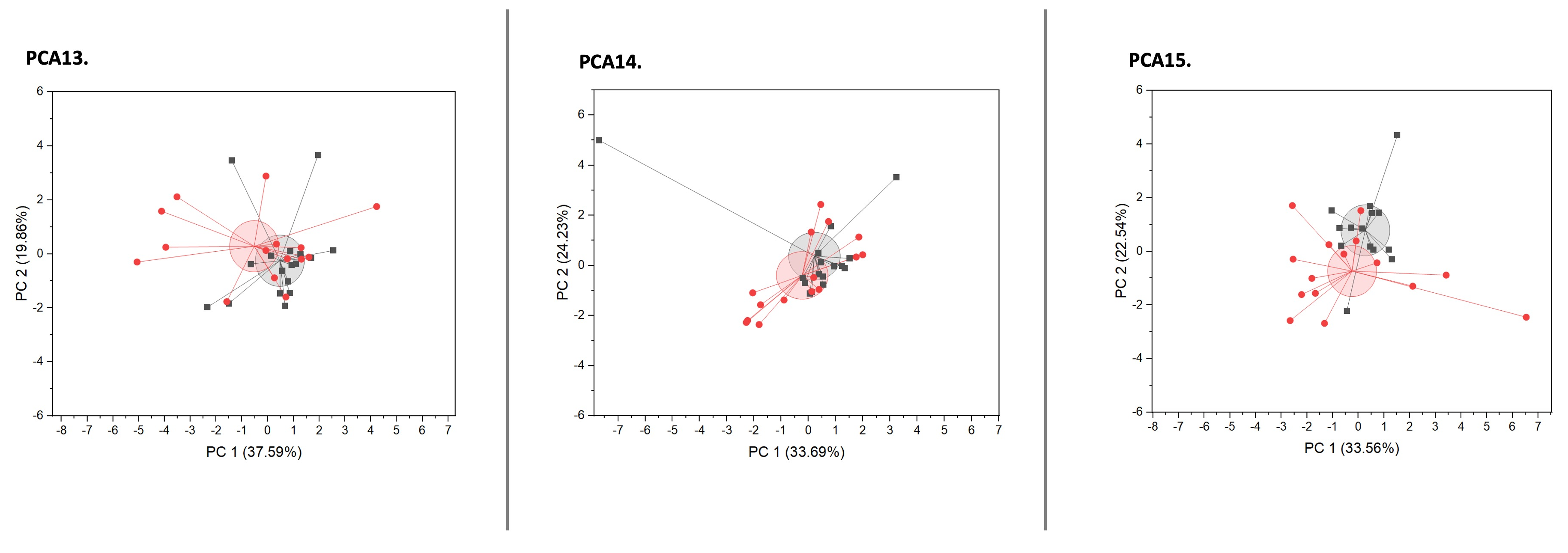


**PCA 13.** B cells in both groups before vaccination.; **PCA 14**. B cells in both groups after the 1^st^ dose of vaccine.; **PCA 15**. B cells in both groups after the 2^nd^ dose of vaccine. Each dot represents a single sample colored by group (gray - IPF, red – control).

**NK cells**

NK cells that allowed discriminating between study and control groups before and after the 2^nd^ vaccine dose displayed CD3-CD56+CD16+ and CD3-CD56brightCD16- phenotypes. After the 1^st^ vaccine dose CD3-CD56+CD16+CD57+ cells also differentiated between analyzed groups.


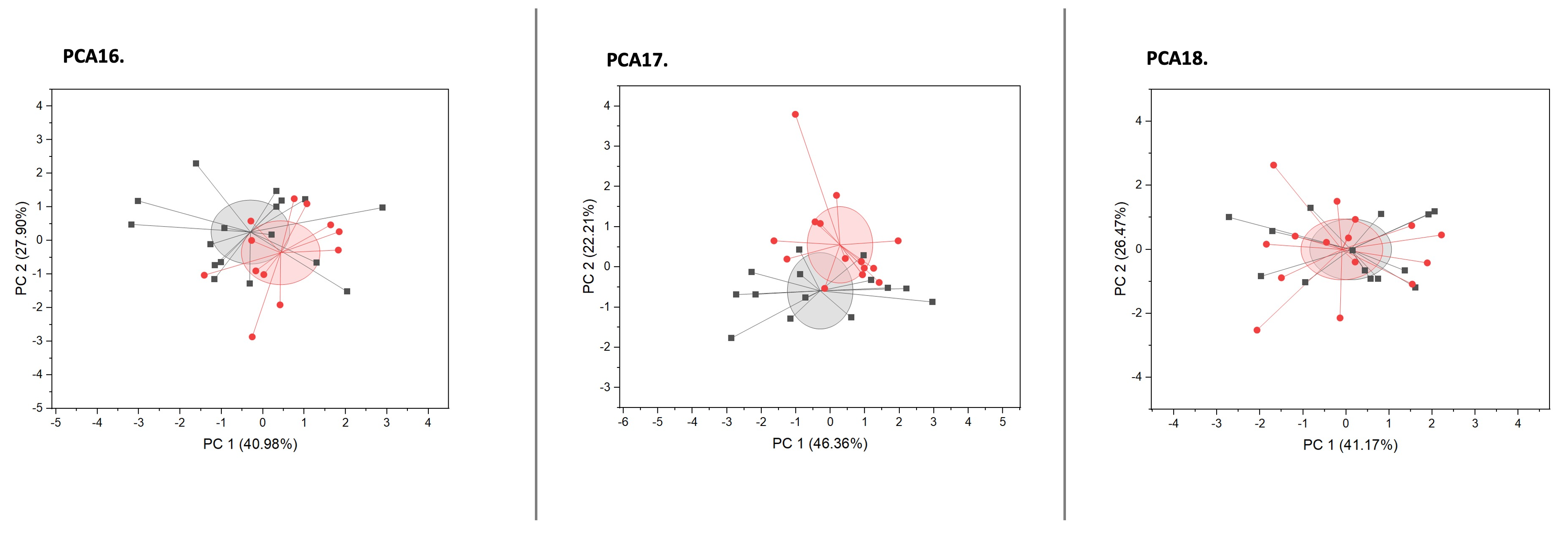


**PCA 16.** NK cells in both groups before vaccination.; **PCA 17**. NK cells in both groups after the 1^st^ dose of vaccine.; **PCA 18**. NK cells in both groups after the 2^nd^ dose of vaccine. Each dot represents a single sample colored by group (gray - IPF, red – control).
